# Ignition of the southern Atlantic seafloor spreading machine without hot-mantle booster

**DOI:** 10.1038/s41598-023-28364-y

**Published:** 2023-01-21

**Authors:** Daniel Sauter, Gianreto Manatschal, Nick Kusznir, Charles Masquelet, Philippe Werner, Marc Ulrich, Paul Bellingham, Dieter Franke, Julia Autin

**Affiliations:** 1 Institut Terre et Environnement de Strasbourg (ITES), Université de Strasbourg, 5 Rue Descartes, 67084 Strasbourg, France; 2 School of Environmental Sciences, Liverpool University, Liverpool, L69 3GP UK; 3 Institut des Sciences de la Terre de Paris (ISTeP), Sorbonne Université, 4 Place Jussieu, 75005 Paris, France; 4 ION-GXT, 31 Windsor Street, Chertsey, Surrey, KT16 8AT UK; 5 Bundesanstalt für Geowissenschaften und Rohstoffe, Geozentrum Hannover, Stilleweg 2, 30655 Hannover, Deutschland

**Keywords:** Geodynamics, Tectonics

## Abstract

The source of massive magma production at volcanic rifted margins remains strongly disputed since the first observations of thick lava piles in the 1980s. However, volumes of extruded and intruded melt products within rifted continental crust are still not accurately resolved using geophysical methods. Here we investigate the magma budget alongside the South Atlantic margins, at the onset of seafloor spreading, using high-quality seismic reflection profiles to accurately estimate the oceanic crustal thickness. We show that, along ~ 75% of the length of the Early-Cretaceous initial spreading centre, the crustal thickness is similar to regular oceanic thickness with an age > 100 Ma away from hot spots. Thus, most of the southernmost Atlantic Ocean opened without anomalously hot mantle, high magma supply being restricted to the Walvis Ridge area. We suggest that alternative explanations other than a hotter mantle should be favoured to explain the thick magmatic layer of seaward dipping reflectors landward of the initial mid-oceanic ridge.

## Introduction

The opening of the South Atlantic, between the Florianopolis and Agulhas-Falkland Fracture Zones (FZs), has often been presented as a case study of continental breakup across a mantle plume^1,2^. The rising Tristan hot plume is thought to have impacted the western Gondwana lithosphere (triggering the Paraná-Etendeka continental flood basalts) and further weakening and thinning it to ultimately lead to the breakup^3^. However, seafloor spreading started > 2000 km south of the Paraná-Etendeka igneous province and propagated northward^4^, reaching the Walvis Ridge, the inferred plume tail, in Aptian times^5^ well after the last sporadic continental flood basalt events at ~ 120 Ma^3^. This northward unzipping of the South Atlantic, starting far away from the plume head, led to question the triggering role of the Tristan plume impingement^4,6,7^. Alternatively, it was proposed that breakup was driven by far-field extensional forces resulting in distributed extension with magmatism occurring as a consequence of decompression melting^6^. The volume of magma would then be a function of one or more factors such as extensional rate, lithospheric thickness mantle temperature, fertility or water content of the inherited mantle.

**Figure 1:**
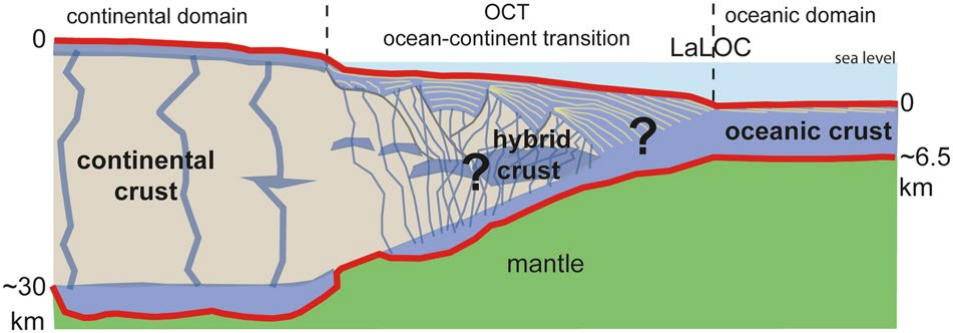
Cartoon highlighting the architecture of the transition between continental domains and unambiguous oceanic crust at magma-rich margins. The nature of the crust in the OCT is highly debated. While seaward dipping reflectors (yellow lines) are easily recognized on seismic reflection images, the amount of magma products (in blue) in the OCT is unknown and fully magmatic crust^14^ as well as exhumed continental crust^16^ are inferred. Here we do not speculate on the nature of this transitional hybrid crust that might be highly intruded. We rather defined the landward limit of the oceanic crust (LaLOC) based on two indisputable reflectors observed on seismic reflection images: the top of basement and the Moho (red lines). Leaving the oceanic domain, the LaLOC is located where the top of the crust shallows while the base of the crust deepens. These two inflexion points mark the departure from the typical geometry of the oceanic crust, with the top of basement broadly parallel to the Moho, testifying steady-state seafloor spreading.

**Figure 2:**
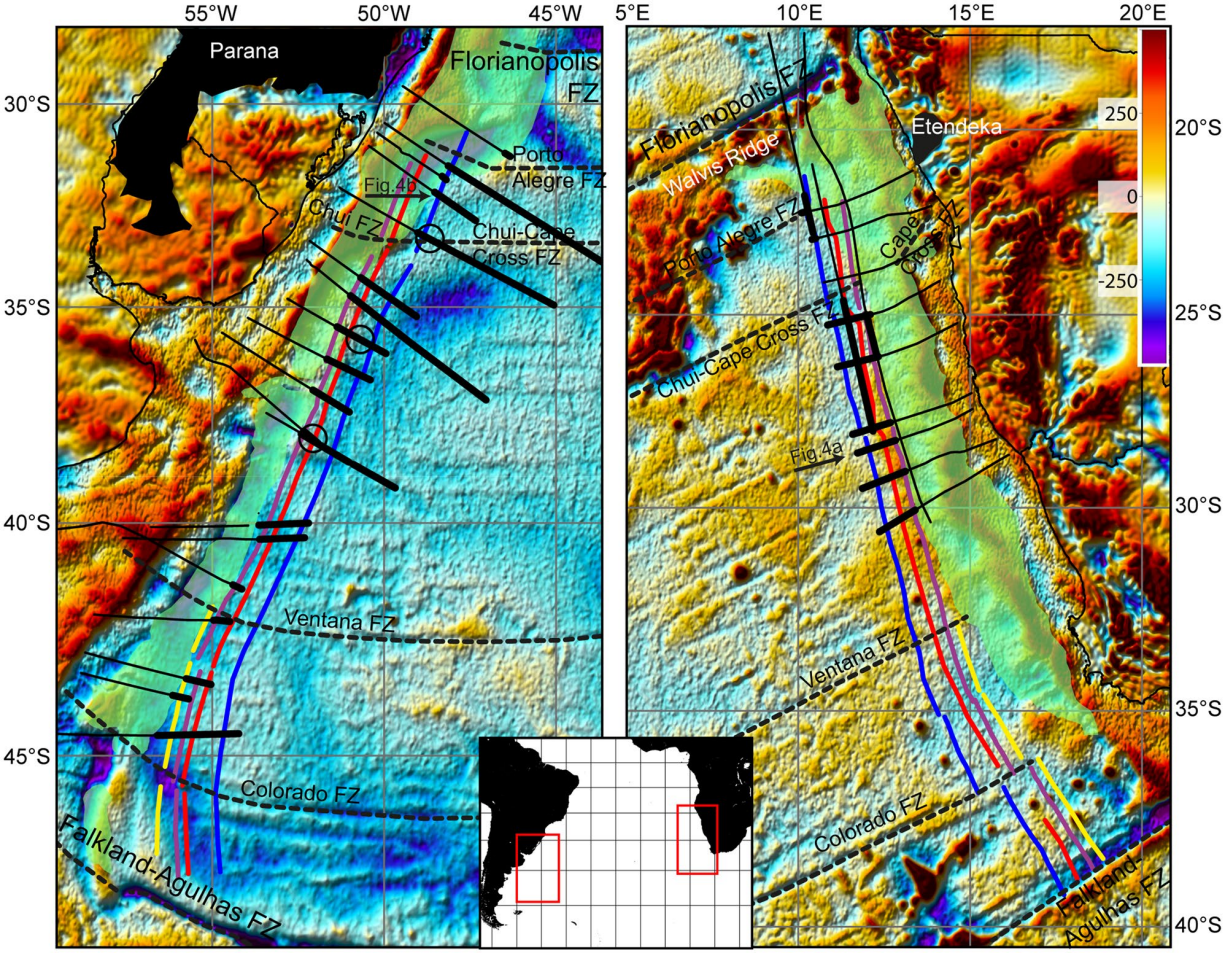
Maps of the conjugated South American (left) and West African (right) volcanic rifted margins. Thin black lines indicate the seismic reflection profiles used in this study (see Supplementary Figs. S1–S3). The thicker lines indicate the occurrence of oceanic crust starting from the landward limit of the oceanic crust (LaLOC) along these seismic reflection profiles. Blue, red, purple and yellow lines are M0.y, M2.o, M3.o and M4.o isochrones, respectively^30^. The black dashed lines indicate fracture zones, the black areas show the Paranà-Etendka igneous province and the green areas show the SDRs extent^39^. The free air gravity anomaly grid (mGal) deduced from satellite altimetry data^58^ is shown in background. Empty circles indicate the location of the three seismic profiles, within the oceanic domain, shown in Fig. 3.

Volcanic rifted margins are typically associated with a thick magmatic layer of seaward dipping reflectors (SDRs)^8^. These large melt volumes were often interpreted as mainly due to melting of anomalously hot sublithospheric material, from the 1980s to present day^1,9^. Although hot plume-sourced mantle is not the unique ingredient, it is conventionally considered as the key one to create SDRs during rifting, and thicker than average oceanic crust during subsequent seafloor spreading^9^. The volume of magma has been used early on to estimate the mantle thermal anomaly thought to be responsible for this excess melt production^1^. However, one of the main pitfalls of this approach lies precisely in determining the volume of magma at volcanic rifted margins^10^. At such margins, continental crust might be highly intruded resulting in a hybrid crust within the ocean-continent transition (OCT)^11^ (Fig. 1). Because intrusions into thinned continental crust cannot be properly resolved using either seismic reflection or refraction techniques, a recent attempt choose to compare magma volumes between seismic profiles, rather than to estimate absolute volumes^12^. Seismic reflection profiles may be subject to multiple geological interpretations with possibly conflicting views: the same transitional crust including SDRs (Fig. 1) may be considered as resulting from seafloor spreading^13–15^ or from the exhumation of middle-lower continental crust^16^. Likewise, crustal refraction velocities and densities across the São Paulo plateau (southeastern Brazilian margin), considered to indicate continental basement, have been re-interpreted as thickened magmatic crust^17^. By contrast, the seismic crustal thickness within the oceanic domain is a proper and widely used estimation of the melt supply per increment of plate separation^18^. It can be readily obtained by picking the top and base (oceanic Moho) of the magmatic oceanic crust on seismic reflection profiles^19^. However, this approach requires access to high-quality deep-penetrating seismic profiles. Here we use 24 such profiles provided to us by ION Geophysical™, south of the Florianopolis FZ (see “Method” Section; Fig. 2 and Supplementary Figs. S1–S3) to investigate how much melt was produced at the onset of seafloor spreading, after the formation of the SDRs and the complete rupturing of the continental lithosphere. This data set was completed by published seismic reflection profiles of the southernmost part of the South Atlantic (see “Method” Section and Supplementary Figs. S1–S3). Our approach is similar to a previous one^13^ but differs in our new way of locating the landward limit of the oceanic crust on both conjugated margins.

## Results

### Locating the landward limit of the oceanic crust

**Figure 3:**
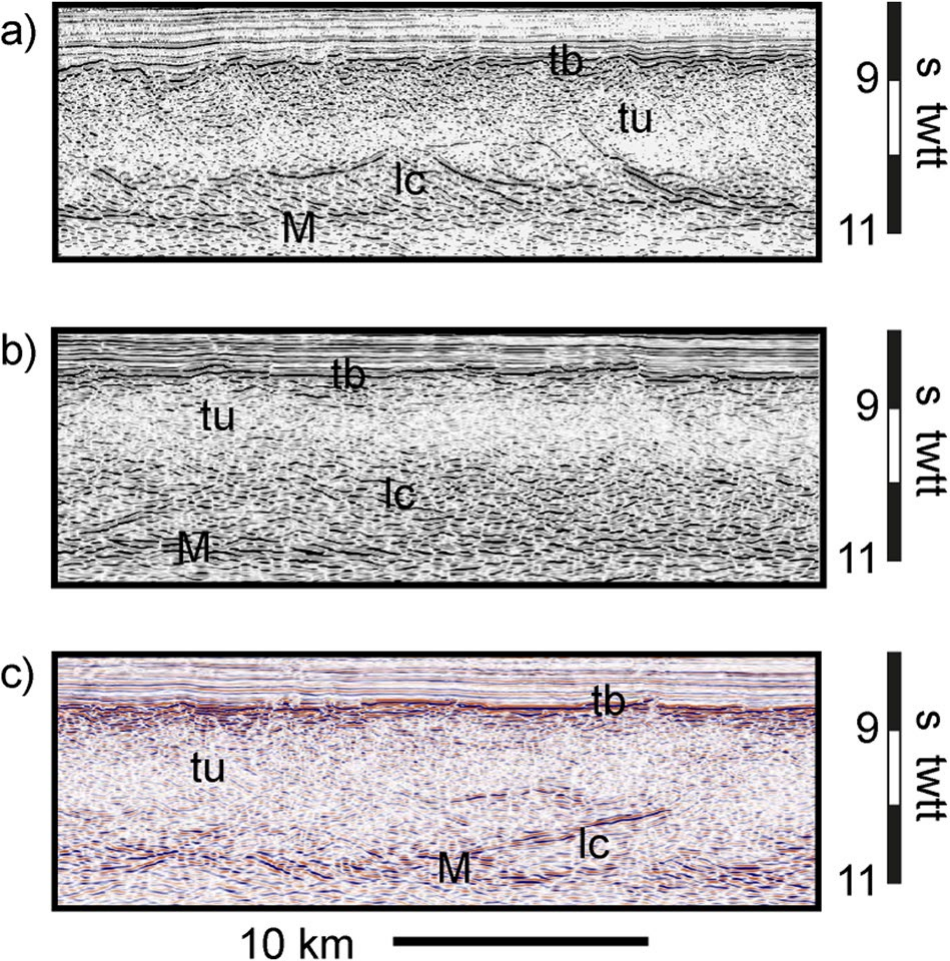
Seismic structure of the oceanic crust for three profiles alongside the South American margin (SAM5, SAM8 and SAM11 from top to bottom). tb indicates the reflective top of basement, tu the transparent unit in the upper crust, lc the more reflective lower crust and M the set of horizontal reflectors interpreted as the oceanic Moho at the base of the crust. The location of these profiles is shown in Fig. 2 (the numbering of the profiles is increasing southward). Courtesy of ION Geophysical.

**Figure 4:**
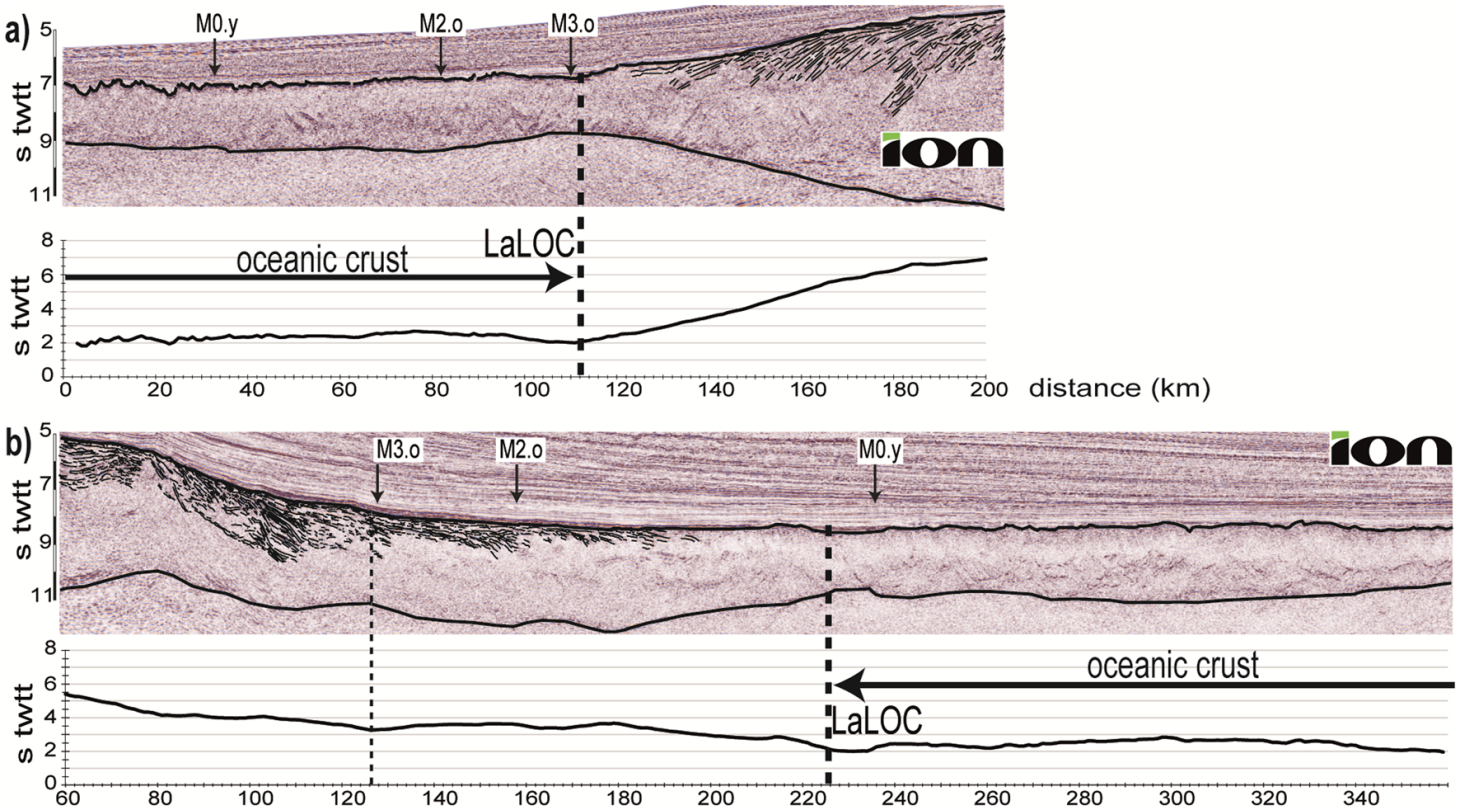
Crustal thickness variations (bottom panel) across the Landward Limit of Oceanic Crust (LaLOC) for two seismic reflection profiles (top panel) at the West African margin (**a**) and the South American margin (**b**). Vertical exaggeration of the seismic reflection profiles: ~ 2x (see Fig. 2 for location, the two lines are not conjugated). Thick black lines indicate the top of basement and Moho while thin black lines show SDRs in the seismic reflection profiles. Courtesy of ION Geophysical.

Mapping the Continent-Ocean Boundary (COB) using different criteria and datasets usually do not correlate to the same location revealing the ambiguity of COB definitions^20^. Here we use the “landward limit of the oceanic crust” (LaLOC) that was previously introduced on both magma poor^21^ and volcanic rifted margins^22^. However, instead of using the seaward edge of SDRs^22^, that may be found locally in oceanic crust^23^, we base our work on the remarkably constant structure and thickness of oceanic crust formed by steady-state seafloor-spreading at mid-ocean ridges (Fig. 1). At fast spreading ridges, as well as at magma-rich intermediate to slow spreading ridges, along flow lines, the Moho is broadly parallel to the top of the crust (at tens of kilometres scale^18^) indicating that oceanic accretion is a steady-state process. In such tectonic settings, the top of basement is generally smooth^24,25^. Alongside the South Atlantic margins, we observe such a well-defined magmatic crust, characterized by a smooth flat top of basement together with a set of horizontal reflectors at the base of the crust. There, the seismic structure is typical of magmatic oceanic crust (i.e. the 1972 Penrose Conference layered oceanic crust model)^26,27^ with, from top to bottom the following successive seismic facies : (1) a reflective top of basement (tb in Fig. 3) with some short reflections in the shallowest part of the crust, (2) a transparent unit in the upper crust (tu in Fig. 3), (3) a more reflective lower crust with dipping structures (lc in Fig. 3) and (4) a set of horizontal reflectors interpreted as the oceanic Moho at the base of the crust (M in Fig. 3). This seismic structure is similar to the one of the oceanic crust observed in the Enderby basin, which opened with slow to intermediate spreading rates (15–60 km/Ma)^28,29^ equivalent to the initial spreading rates in the South Atlantic (30–55 km/Ma)^30–33^ (Fig. S4). Leaving this oceanic domain toward the continents, either (1) the top of basement sharply shallows as the Moho strongly deepens creating a crustal taper and a sudden crustal thickening up to ~ 9 s TWTT (Fig. 4a), or, (2) the top of basement and Moho diverge for ~ 40 km, resulting in an up to ~ 3–5 s TWTT thick crust whose top and base show then little depth variation for the next 60–70 km before strongly diverging continent-ward (Fig. 4b). We define the first continent-ward occurrence of such inflexion points at both top and base of the crust as the LaLOC arguing that any significant departure of the common geometry of the ocean crust marks a rupture in the stable magma production, which characterizes steady-state seafloor spreading in the zone of plate divergence that is durably localized^34^. Although we used seismic profiles, which are mostly perpendicular to both margins, some are crossing fracture zones, produced by transform faults of the initial spreading centre. We were thus careful to disregard the along-axis crustal thickness variations resulting from this first-order segmentation of the initial mid-oceanic ridge. We note that at magma-rich spreading centres, between transform faults, the thermal regime is stable and hot enough to allow melt re-distribution within the crust, leading to almost constant crustal thickness, both along and across the spreading axis^35^. Landward of the LaLOC, both the crustal production via SDRs, and the hydrothermal cooling, through large faulting, change dramatically. While we do not preclude magmatic crust continent-ward of the LaLOC (Fig. 1) its seaward thickness decrease could not result from stable seafloor spreading. Therefore, in this paper, we are focusing on unambiguous Penrose-type oceanic crust, which starts at the LaLOC and indicates undeniable steady-state seafloor spreading at the initial South Atlantic mid-oceanic ridge (Fig. 2). We stress that our way to define the LaLOC is very conservative as it is based on the geometry of two indisputable reflectors on seismic reflections profiles (the top of basement and Moho) rather than on non-unique interpretations of the nature of the crust within the OCT (Fig. 1).

### Timing of the onset of steady-state seafloor spreading in the South Atlantic

**Figure 5:**
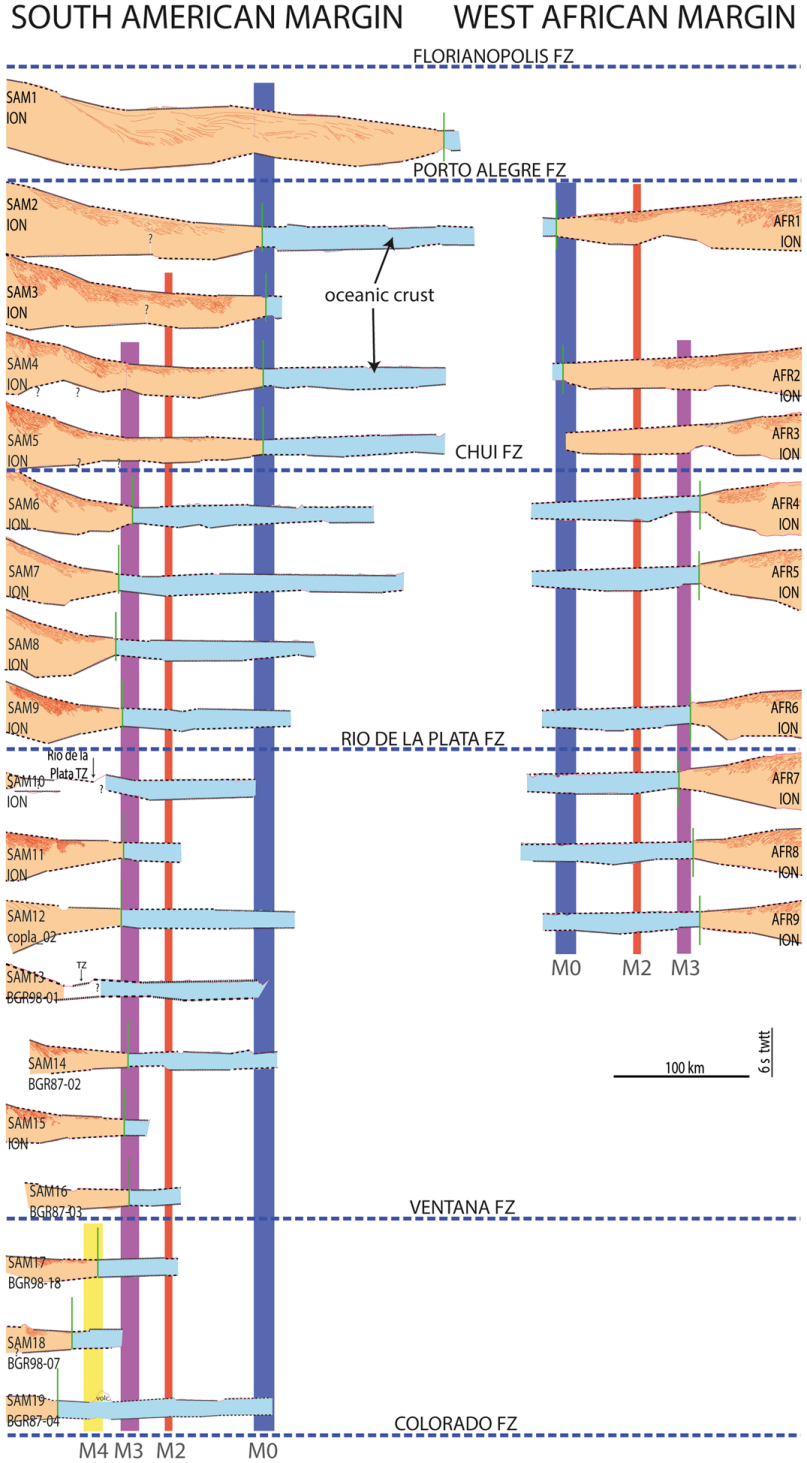
Synthetic view of all profiles used in this study. Light blue indicates oceanic crust. Orange indicates undifferentiated intruded and thinned crust, which may change gradually oceanward from unthinned continental crust up to highly intruded crust (hybrid crust) but is sketched here as a single type of crust for simplicity. Thin red line indicate SDRs. Vertical exaggeration of the seismic reflection profiles: ~ 2x. Seismic lines are aligned with respect to M2.o isochrone (vertical red line). Blue, red, purple and yellow vertical lines indicate M0.y, M2.o, M3.o and M4.o isochrones, respectively^30^. The blue dashed lines indicate fracture zones^39^.

Placing the LaLOC on the most recent map of the M4.o–M0.y magnetic isochrones^30^ (127.5 and 121 Ma respectively using the time scale of Ogg^36^, see Table S1 and Figs. 2, 5, S5) confirms that seafloor spreading initiated earlier than M4.o time north of the Colorado FZ and that the onset successively was delayed northward to about M3.o time (126.5 Ma) at the Ventana FZ^37^. Our most striking finding is that, further north, the LaLOC lies close to the M3.o isochrone for ~ 1000 km up to the Chui-Cape Cross FZ (Figs. 2, 5). From this FZ up to the Porto Alegre FZ, the onset of seafloor spreading is dated at about M0.y time. North of the Porto Alegre FZ there is only one seismic line reaching unequivocal oceanic crust^5^ and the LaLOC falls in the Cretaceous Magnetic Quiet Zone, with an estimated age of ~ 116 Ma (assuming a 25 km/Myr half rate^32^). Seafloor spreading propagated thus northward, starting ~ 130.3 Ma ago (M9r.o) close to the Agulhas-Falkland FZ^31^ and reaching south of the Florianopolis FZ ~ 116 Ma ago but, with a faster propagation rate along nearly half of this segment length at M3.o time. Initial seafloor spreading was intermediate (~ 50–55 km/Myr full rate) during the time of magnetic chrons M9r.o to M3.o to slow (30–45 km/Myr) from M3.o to M0.y^30–33^.

### Oceanic crustal thickness variations at the initial South Atlantic spreading centre

The oceanic Moho is found 2.08 ± 0.15 s TWTT on average beneath the top of basement at the LaLOC corresponding to a 6.65 ± 0.47 km mean crustal thickness (see “Method” Section and Table S2). This mean thickness at the LaLOC is similar to the 6.62 ± 0.86 km average calculated from the worldwide compilation of Christeson et al.^38^ for > 100 Ma old oceanic crusts away from hotspots (see “Method” Section). Our crustal thickness values at the LaLOC are at odds with previous estimations that are up to 3 km larger^13^. These previous estimations were obtained using a LaLOC defined by a combination of magnetic and seismic characteristics and allowing the LaLOC to coincide with overlying SDRs^13^. This discrepancy reveals the dependency on the definition and thus the location of the LaLOC (Fig. S6). Interestingly, our LaLOC falls close to a recent mapping of the seaward edge of SDRs^39^ (see the green area in Fig. 2), a criteria that was originally used to define the landward limit of unambiguous oceanic crust^22^. Moreover, our crustal thickness estimations are remarkably consistent along both the South American and African margins supporting the robustness of our method (Table S2). Therefore, we conclude that the magma supply at the initial South Atlantic spreading centre is not different from that at the mid-oceanic ridge system worldwide at Early Cretaceous times.

**Figure 6:**
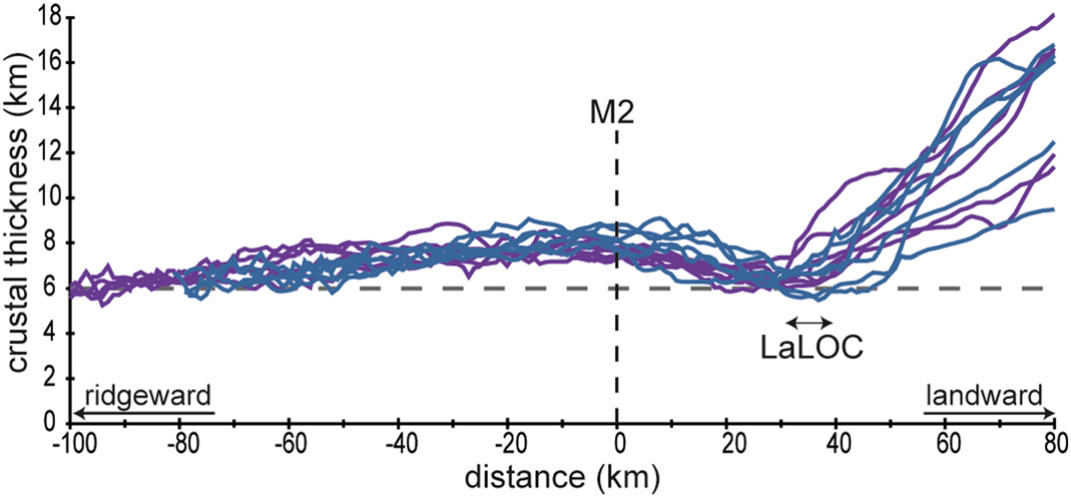
Crustal thickness variation with respect to the M2.o magnetic anomaly on both conjugated margins between Chui-Cape Cross FZ and 38°S on the South American plate and 31°S on the African plate for 6 profiles across the Namibian margin (in dark blue) and 7 profiles across the Uruguayan and Brazilian margins (in purple). Distances are increasing landward. The dashed horizontal grey line indicates 6 km crustal thickness.

Although the top and the base of the crust are broadly parallel at long distances, the thickness of the oceanic crust may change gradually toward anomalously hot/cold or depleted/enriched mantle sources^40^. While little thickness variations are observed in the southernmost part of the study area, we notice a small but systematic variation from the LaLOC (dated at M3.o time) in a seaward direction on both conjugated margins, for ~ 700 km south of the Chui-Cape Cross FZ (Fig. 6). There, the crustal thickness first increases, reaching 7–9 km at ~ 40 km from the LaLOC (i.e., at ~ M2.o time; 124.7 Ma), and then decreases again, coming back to average oceanic crustal thickness (~ 6.5 km) at ~ 120 km from the LaLOC (somewhat earlier than M0.y time) (Fig. 6). North of the Chui-Cape Cross FZ, oceanic crust does not show such systematic thickness variations. However, there, continent-ward of the LaLOC (dated at M0.y time), the crust thickens gradually for ~ 40 km, shows smaller variations for at least ~ 60 km before increasing more strongly continent-ward at M3.o time north of Chui-Cape Cross FZ and somewhat later northward, south of the Porto Alegre FZ (Figs. 5, S7). Therefore, while M3.o marks the onset of steady-state spreading to the south of the Chui-Cape Cross FZ, a sharp change in magma supply and/or tectonic thinning also happened at exactly that time, affecting an area continent-ward of the LaLOC to the north of this FZ. Moreover, we note a gradual change of the seismic structure from a typical oceanic crust to the south of this FZ to a thicker crust with increasing large SDR wedges northward (> 2 s TWTT thick) after M3.o time (Figs. 5, S8). We therefore suggest that, along both conjugated margins, there is a transition from average oceanic crust to a northward thickening igneous crust, both being formed simultaneously from M3.o time on. While we cannot exclude that this thick igneous crust may be a hybrid crust including remnants of continental basement^5,11,39^, the occurrence of large SDR wedges (30–40 km wide and 8 km thick) argues for a primarily magmatic origin for the crust between M0.y and ~ M3.o north of Chui-Cape Cross FZ^5,39^. This is also in line with the observed landward shift of the high-velocity lower crust (HVLC) identified by refraction seismology, that may correspond to underplated and intruded mantle-derived magmas formed at the end of the lithospheric breakup process^41^. Close to the Walvis Ridge, and at the conjugated South American margin, large volumes of HVLC extend far seaward of the inner SDRs while, southward, the HVLC is below the inner SDR wedges^41^.

**Figure 7:**
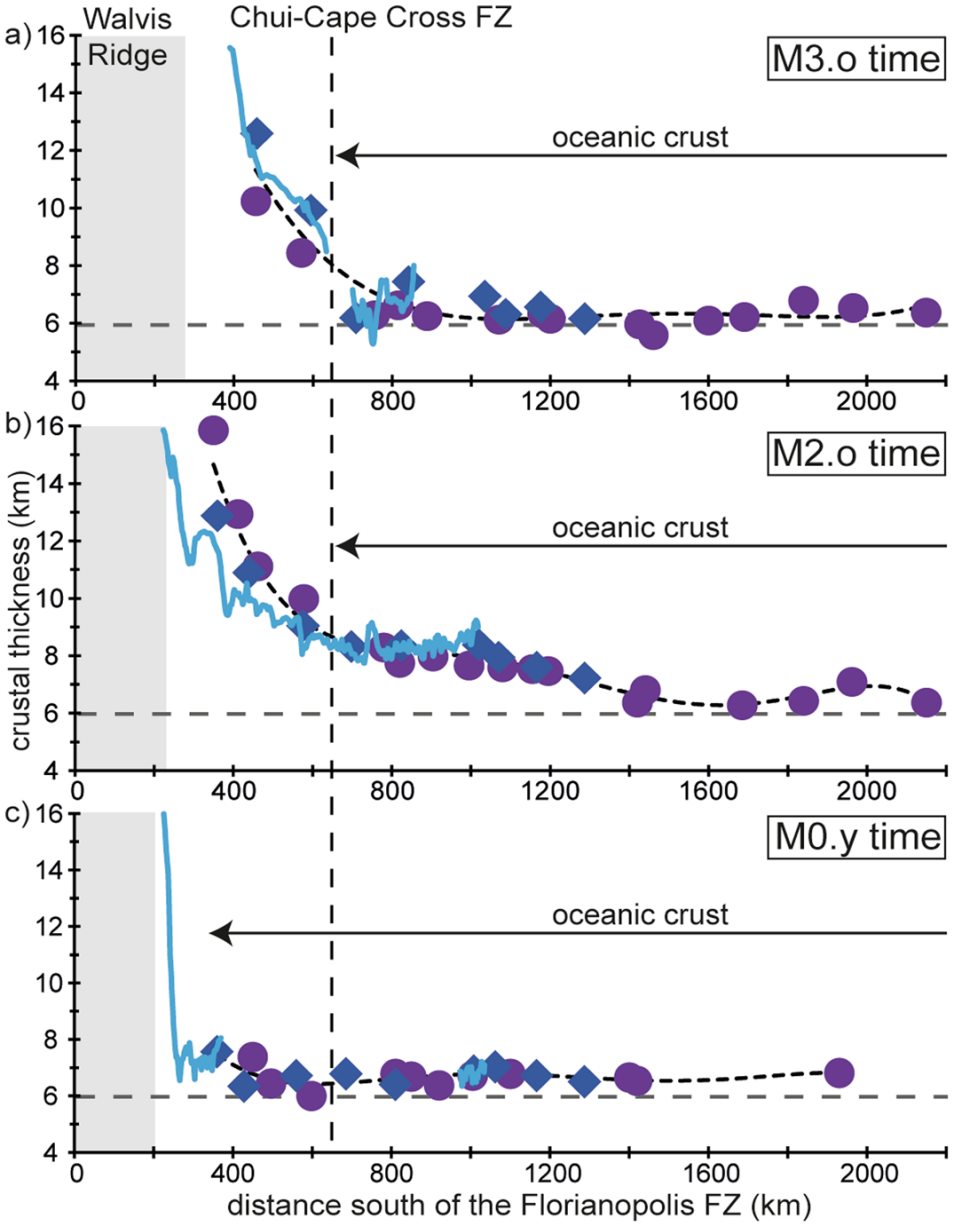
Crustal thickness variation along M0.y, M2.o and M3.o isochrones alongside both conjugated margins with respect to the Florianopolis FZ. Purple circles are from the South American plate and dark blue lozenges are from the African plate. Black dashed lines indicate the best polynomial fits. The blue lines show crustal thickness variations from seismic strike lines along the Namibian margin close to the isochrones (< 20 km). The dashed horizontal grey line indicates 6 km crustal thickness. The grey shaded areas correspond to the Walvis ridge width taken from the free air gravity anomaly map derived from satellite altimetry data.

This northward evolution is best shown by the crustal thickness variations along the M0.y, M2.o and M3.o isochrones plotted against distance increasing southward from the Florianopolis FZ (Fig. 7). At M3.o time, the thickness of the oceanic crust is normal up to the Chui-Cape Cross FZ, i.e. along ~ 75% of the segment length between the Agulhas–Falkland and the Florianopolis FZs (~ 2300 km). To the north of the Chui-Cape Cross FZ, the crustal thickness increases strongly toward the Walvis Ridge, the inferred plume tail of the Tristan hotspot (Figs. 7a, S8). At M2.o time, normal oceanic crustal thickness is only observed far to the South and more than ~ 1600 km away from the Florianopolis FZ (Fig. 7b). From this southernmost part the crust thickens gradually northward up to the Chui-Cape Cross FZ and then more strongly toward the Walvis Ridge. At M0.y time, normal oceanic crust is observed all along the conjugated margins except close to the Walvis Ridge (Fig. 7c). Northward thicker crust is thus found from ~ 600 km, ~ 1600 km and ~ 300 km away from the Florianopolis FZ at M3.o, M2.o, and M0.y times, respectively. We discuss the evolution of this northward increasing magma supply in the next section.

## Discussion

### Was there an interaction between the Paraná–Etendeka igneous province and the initial south Atlantic ridge?

**Figure 8:**
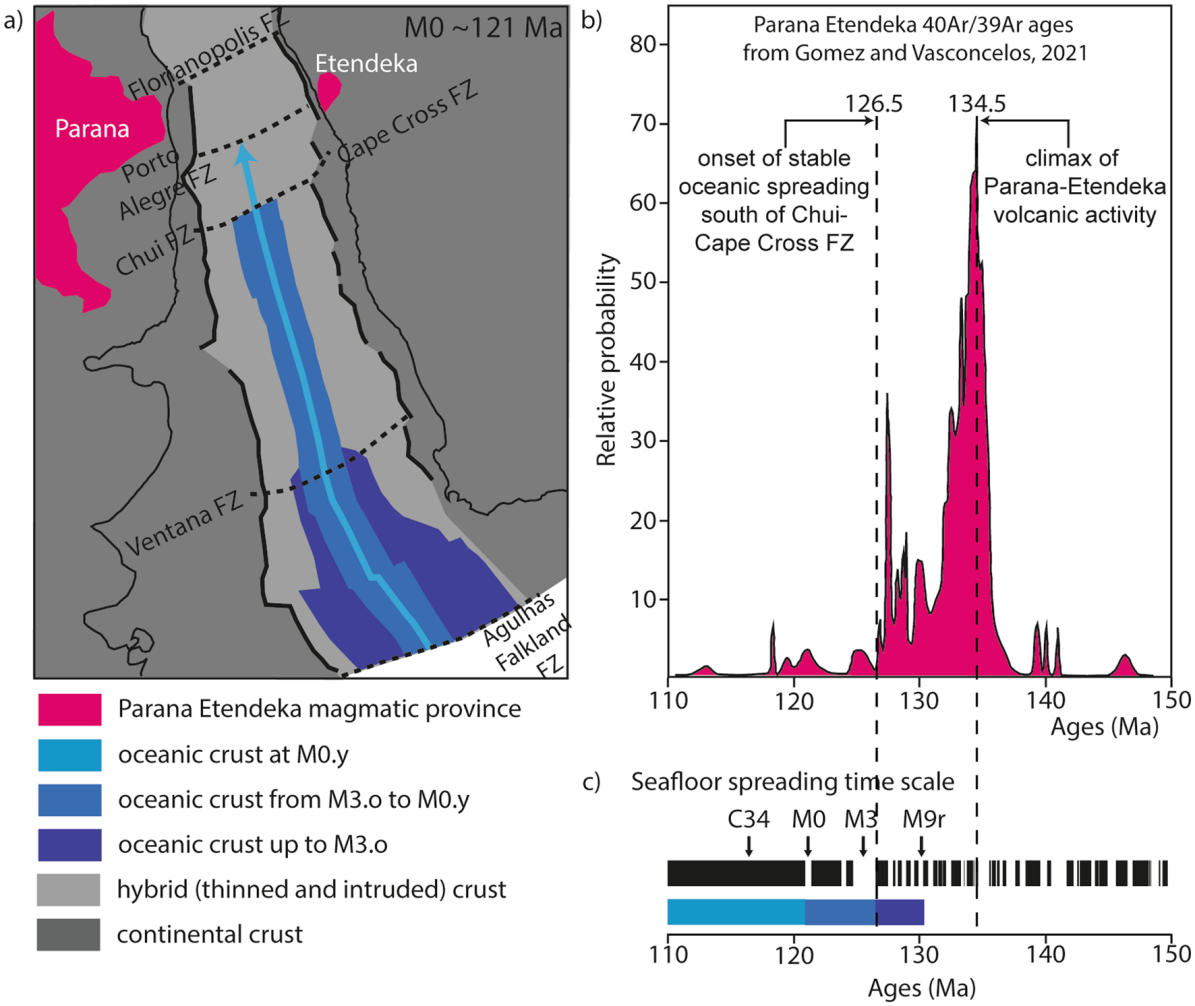
Onset of steady-state seafloor spreading in the South Atlantic Ocean relative to the Paraná–Etendeka volcanic event. (**a**) map reconstructed at M0, (**b**) probability for radiometric dated Paraná and Etendeka volcanic events between 110 and 150 Ma^42^, (**c**) geomagnetic time scale between 110 and 150 Ma and corresponding periods of seafloor spreading shown in a).

Continental flood basalt volcanic activity began at ~ 145 Ma, climaxed at 134.5 Ma and evolved from the Paraná plateau to Etendeka and southern Angola (134–132 Ma)^3,42^. Seafloor spreading started, at M9r.o time (130.3 Ma) ~ 2000 km away, close to the Agulhas–Falkland FZ^31^, while volcanic activity had already declined at the Paraná–Etendeka igneous province (Fig. 8). The initial mid-oceanic ridge propagated northward in the southernmost part of the South Atlantic, with a normal melt supply resulting in average crustal thickness during initial seafloor spreading. Continental flood basalt activity then decreased even more in the Paraná–Etendeka igneous province and steady-state seafloor spreading began, still with a normal melt supply, at M3.o time (126.5 Ma), along a ~ 1000 km long segment between the Ventana and the Chui-Cape Cross FZs. At that time, increasing volume of magma is erupted north of the Chui-Cape Cross FZ, approaching the Paraná-Etendeka igneous province. In agreement with the opening of the South Atlantic by successive northward unzipping^4,37,43^, ridge propagation stopped or strongly slowed down temporarily before starting again northward at M0.y time (121 Ma) while the last sporadic flood basalt episodes continued until ~ 120 Ma leading to the Walvis Ridge volcanism^3^ (Fig. 8). At M2.o time (124.7 Ma), however, thicker than average oceanic crust was formed along the initial ridge up to ~ 1000 km south of the Chui-Cape Cross FZ. This is surprizing given that oceanic crustal thickness in exactly this area did not deviate from average values both before M3.o and after M0.y times. This means that the magma supply at the onset of steady-state seafloor spreading was normal, but increased substantially during M2.o time while the spreading rate remained unchanged. We hypothesize that such larger melt supply along the initial ridge axis, culminating at M2.o time, results from the interaction of spreading with the declining Paraná–Etendeka igneous province while there is no evidence for such interaction south of the Chui-Cape Cross FZ at M3.o and after M0.y times. Assuming that increasing the mantle potential temperature by 12.5 °C increases the oceanic crustal thickness by 1 km^44^, it may be speculated that the radial propagation of a small thermal anomaly (∼25 °C) within an asthenospheric channel beneath the initial South Atlantic ridge axis may have resulted in the observed crustal thickness variation (~ 2 km; Figs. 6, 7). If there was such an asthenospheric elevated temperature or chemical anomaly it certainly was transient (~ 5 Ma), possibly controlled by the decreasing volcanic activity at Paraná–Etendeka. The magma budget along the northern part of the South Atlantic would then be a result from a trade‐off between the activity and the distance to the Paraná–Etendeka igneous province.

### No hot mantle booster needed for the onset of seafloor spreading

As normal thickness crust was formed along the northward propagating initial South Atlantic ridge for ~ 75% of its length up to M3.o time, we conclude that there, seafloor spreading started without anomalously hot or fusible mantle. This is in agreement with seismic refraction studies that did not find traces of largescale intrusions within the continental crust at the junction with the Walvis Ridge and thus concluded that no broad plume head existed during opening of the South Atlantic and that anomalous mantle melting occurred only locally^45,46^.

North of the Chui-Cape Cross FZ, thick crust has been formed between M3.o and M0.y times before the normal melt supply resumed along the initial South Atlantic ridge. This thick igneous crust shows many similarities with Icelandic crust^47^, including large SDR wedges, rift propagations and ridge jumps^39,47^. We therefore suggest that, as in Iceland, crustal accretion in the northern part of the South Atlantic corresponds to spreading at a very high magma supply resulting from the interaction with magmatic activity at the Paraná–Etendeka province. The magmatic supply over tectonic extension ratio is so high that crustal construction corresponds to a complex 3D mode of accretion with significant rift zone-parallel displacements, lava flows subsidence and mass redistributions^47^. As volcanic activity declined in the Paraná–Etendeka province, the magma supply also decreased north of the Chui-Cape Cross FZ giving way to the onset of normal steady-state seafloor spreading at M0.y time in the area up to the Porto Alegre FZ and at ~ 116 Ma south of the Florianopolis FZ. The Chui-Cape Cross FZ may not have acted as a mechanical or thermal barrier for the propagation of the initial ridge as there, the crust thickens northward progressively. Rather, the South Atlantic seafloor spreading machine may have stalled until the magma supply was optimal for an ideal steady state functioning.

Finally, our results may have important implications for the origin of the SDRs in the more proximal parts of the South Atlantic margins and other volcanic rifted margins. Here, we show that there is no anomalously hot mantle beneath the initial South Atlantic spreading ridge away from the Walvis ridge area. One could argue that hot mantle potential temperatures (with a 150–300 °C thermal anomaly relative to the ambient mantle^3,9,13^) still triggered the production of large volumes of melt and SDRs formation along the rifted margins while volcanic activity climaxed in the Paraná–Etendeka province ~ 134.5 Ma ago^39^ and then went back to normal 8 Myrs later (at M3.o time, 126.5 Ma ) at the onset of seafloor spreading south of Chui-Cape Cross FZ (Fig. 8). This mantle potential temperature variation may be compared to the one along the Mid-Atlantic ridge south of Iceland, a classic example of a ridge-centred plume. There, the mantle potential temperature variation inferred to produce the crustal thickness variations along the Reykjanes ridge is an order of magnitude smaller for the same timescale (3–8 Myrs)^48^. This suggests that such a fast cooling of the mantle is rather unlikely. Alternatively, we propose that either the magma volume along the South Atlantic volcanic margins has to be re-evaluated downward and/or explanations other than a hotter mantle, prior to the onset of seafloor spreading, have to be favoured to explain massive magmatic production there. This is in line with recent results from a numerical approach demonstrating that the large volumes of magma at volcanic rifted margins can be explained by depth dependent extension and very moderate excess mantle potential temperature significantly smaller than previously suggested^49^. Other recent numerical models also show that, using the same average mantle potential temperature, but different crustal and mantle thinning rates, results in either magma-poor or magma-rich rifted margin segments, while the final oceanic crustal thickness is similar (ca. ~ 7 km)^50^. Moreover, recent thermobarometric estimates from geochemical data show that the mantle beneath the West Greenland volcanic province was not very hot (~ 1350 °C) but still able to produce a ~ 7 km thick SDR volcanic sequences at the volcanic rifted margin of Baffin Bay^51^. There, observations related to the geometry, spatial and temporal extent of rifting, seafloor spreading and rift-related magmatism do not fit the predictions of a model of breakup in response to the arrival of a mantle plume^52^. Therefore, we suggest that hot plume-sourced mantle, that is conventionally considered as the key parameter to create SDRs during rifting and thicker than average oceanic crust during initial seafloor spreading^1,9^, may not play such a role of booster away of the plume head.

## Methods

Details on ION Geophysical’s global BasinSPAN seismic data library are now available from TGS™ website (https://map.tgs.com/myTGSMap/Data-Library). Kirchhoff prestack time were performed on all seismic surveys following proprietary ION Geophysical processing workflow (example of processing workflow in^53^). PSTM profiles were initially available with a 18 s record length. The reader is referred to previous publications^37,43,54^ for acquisition and processing parameters of other used seismic reflection profiles.

We picked the reflective top of basement and the reflectors at the base of the crust to obtain the crustal thickness in s TWTT that we then convert in kilometres using 6.4 km/s bulk velocity obtained by Hoggard et al.^55^ for more than ~ 30 Ma old oceanic crust by compiling velocity-depth profiles based upon waveform modelling of modern wide-angle experiments (see supporting information of Hoggard et al.^55^). The obtained estimations of oceanic crustal thickness are close to the ones obtained using the relationship of Canales et al.^56^ : y [km] = 3.054x [s TWTT] + 0.261 (Fig. S9). The mean difference between the two estimations is 0.03 km for 1–3 s TWTT. The relationship of Canales et al.^56^, obtained for magmatic oceanic crust at the fast spreading East Pacific Rise, is in fact a refinement of the one of Barth et al.^18^ who first argued that the crustal thickness can be inferred from crustal reflection travel times even in cases where the crustal velocity structure is unknown because there is a linear relationship between the crustal TWTT and the crustal thickness (inferred from the global compilation of White et al.^57^).

We stress that we did not map the oceanic domain using a fixed pre-defined crustal thickness (e.g. 2 s TWTT). By contrast, leaving the oceanic domain, we determine the LaLOC as the first continent-ward occurrence of inflexion points at both the top of the crust, which shallows, and at the base of the crust, which deepens. These inflexion points mark thus the departure from the more or less constant crustal geometry (the top of basement parallel to the Moho) typically found in the oceanic domain (Fig. 1). Mean oceanic crustal thickness at the LaLOC was then calculated along 5 km-long sections ocean-ward of the LaLOC. The 6.65 ± 0.47 km mean oceanic crustal thickness, obtained using all seismic profiles, is similar to the 6.62 ± 0.86 km thickness average for > 100 Ma old oceanic crusts away from hotspots calculated using 23 thickness values of the compiled data set of Christeson et al.^38^ (see the > 100 Ma old oceanic crust thickness values in their supplementary material Data Set S1).

## Supplementary Information

Supplementary Information.

## Data Availability

Thanks to ION™ the seismic data supporting this study are available within the paper and the supplementary information file and can be used as so to reproduce the findings. The raw data are now private properties of TGS™ which have to be contacted for any lending or acquisition (https://www.tgs.com/). The gravity grid of Sandwell et al. (2014) is freely available from the website of the UCSD (topex.ucsd.edu). The global Earth Magnetic Anomaly Grid at 2 arc minute resolution version 3 (EMAG2v3) is freely available from the web site of the NOAA (www.ncei.noaa.gov).
