# Supplementary material for: Ignition of the southern Atlantic seafloor spreading machine without hot-mantle booster
Source: Sci Rep. 2023 Jan 21;13:1195. doi: 10.1038/s41598-023-28364-y (PMC9867738; doi:10.1038/s41598-023-28364-y)
Supplement: Supplementary file 1 — Supplementary Information. [file 41598_2023_28364_MOESM1_ESM.pdf]

SUPPLEMENTARY MATERIAL

Ignition of the Southern Atlantic seafloor spreading machine without hot-mantle booster

Daniel Sauter1*, Gianreto Manatschal1, Nick Kusznir2, Charles Masquelet3, Philippe Werner1, Marc Ulrich1, Paul Bellingham4, Dieter Franke5, Julia Autin1

1) Institut Terre et Environnement de Strasbourg, Université de Strasbourg, CNRS UMR 7063, 5 rue Descartes Strasbourg 67084 France ; daniel.sauter@unistra.fr

2) School of Environmental Sciences, Liverpool University, Liverpool, United Kingdom

3) Sorbonne Université, CNRS, Institut des Sciences de la Terre de Paris (ISTeP), 4 place Jussieu, 75005, Paris, France

4) ION-GXT, 31 Windsor Street, Chertsey, Surrey, KT16 8AT, UK

5) Bundesanstalt für Geowissenschaften und Rohstoffe, Geozentrum Hannover, Stilleweg 2, 30655 Hannover, Deutschland

Nature Scientific Reports

Fig. S1: Location of the seismic reflection profiles used in this study (black lines). Profile names are given next to each line. Profiles crossing the West African margin are shown in supplementary Fig. S2 while profiles crossing the South American margin are shown in supplementary Fig. S3. Thin black lines indicate the seismic reflection profiles and the thicker lines indicate the occurrence of oceanic crust starting from the landward limit of the oceanic crust (LaLOC) along these seismic reflection profiles. Blue, red, purple and yellow lines are M0.y, M2.o, M3.o and M4.o isochrones, respectively, after Collier et al. (2017). The black dashed lines indicating fracture zones, the black areas showing the Parana-Etendka igneous province and the green areas showing the SDRs extend are from Chauvet et al., (2020). The free air gravity anomaly grid deduced from satellite altimetry data from Sandwell et al. (2014) is shown in background.

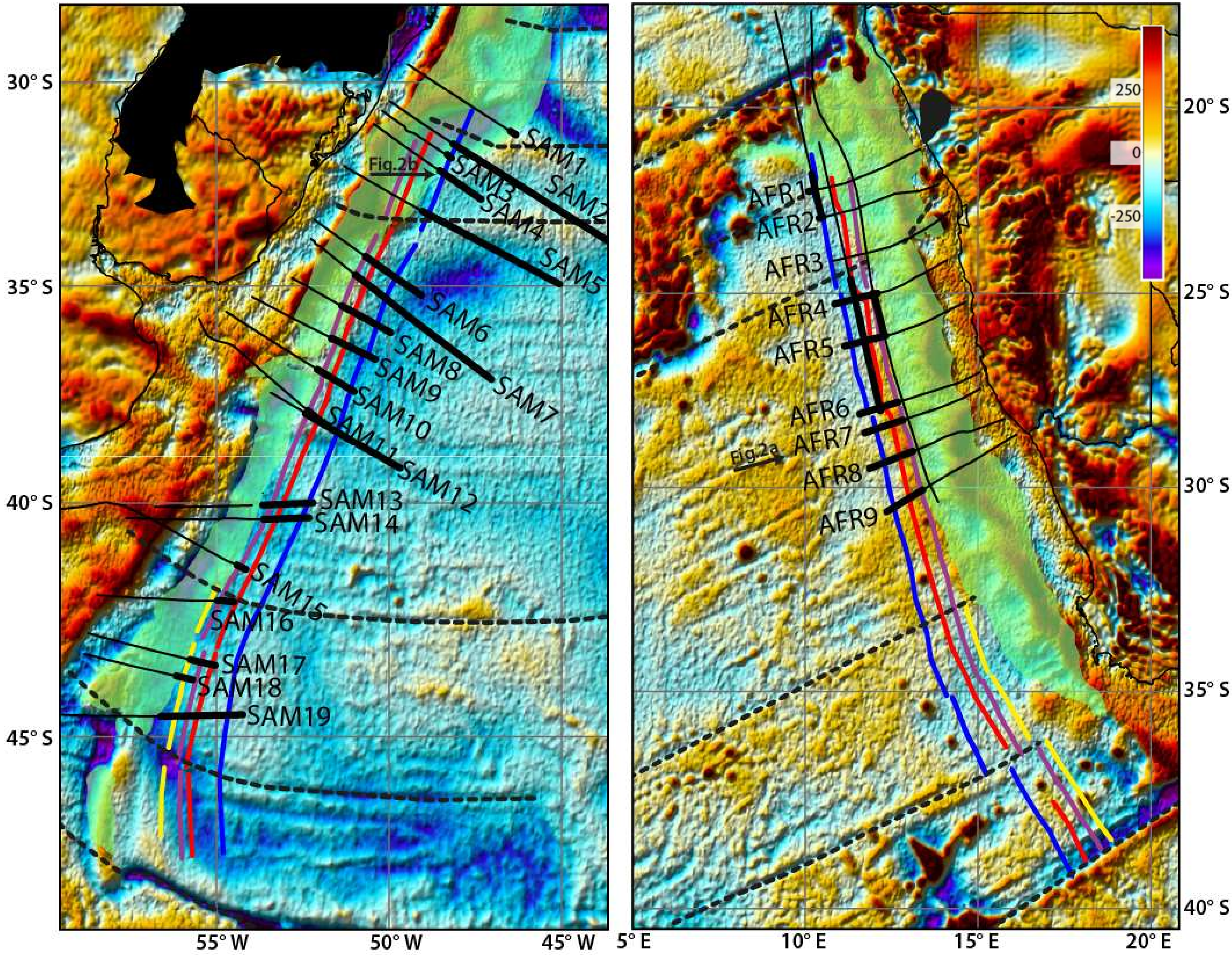

Fig. S2: The next 9 pages show the seismic reflection profiles that we used along the West African margin. Top basement, Moho and SDRs are shown for each profile. The crustal thickness variation is shown below. Green vertical lines are located where top basement shallows while Moho deepens. LaLOC indicates the landward limit of oceanic crust. M4 is for M4.o (127.5 Ma); M3 is for M3.o (126.5 Ma); M2 is M2.o (124.7 Ma) and M0 is for M0.y (121 Ma) and indicate where the profiles cut the isochrones. Courtesy of ION Geophysical.

Profile AFR1

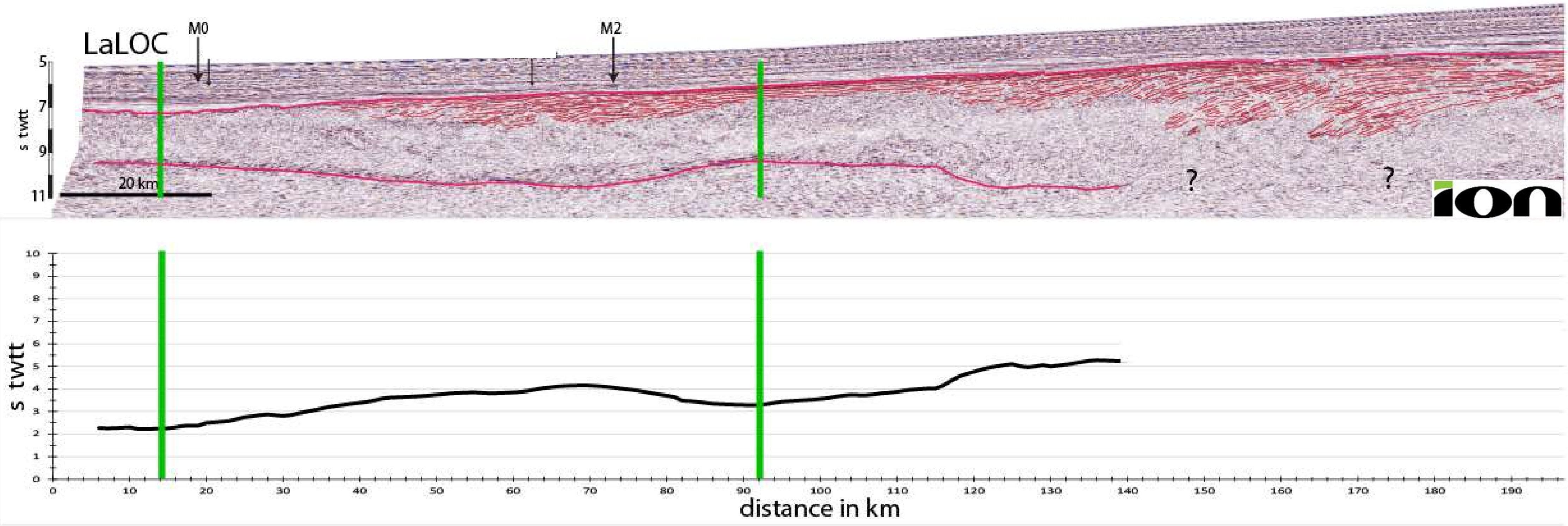

Profile AFR2

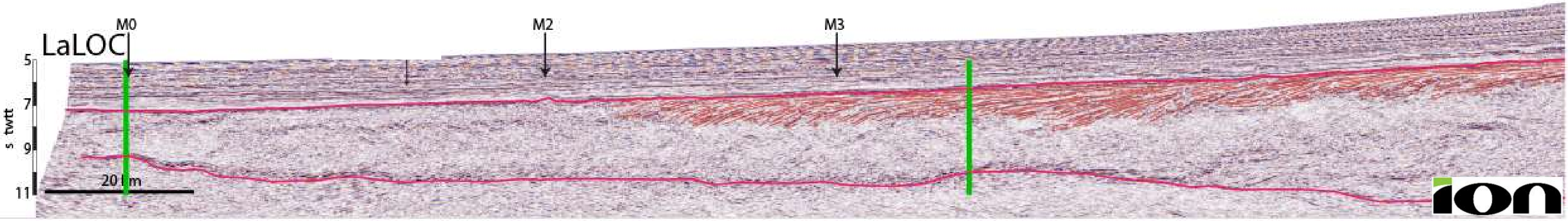

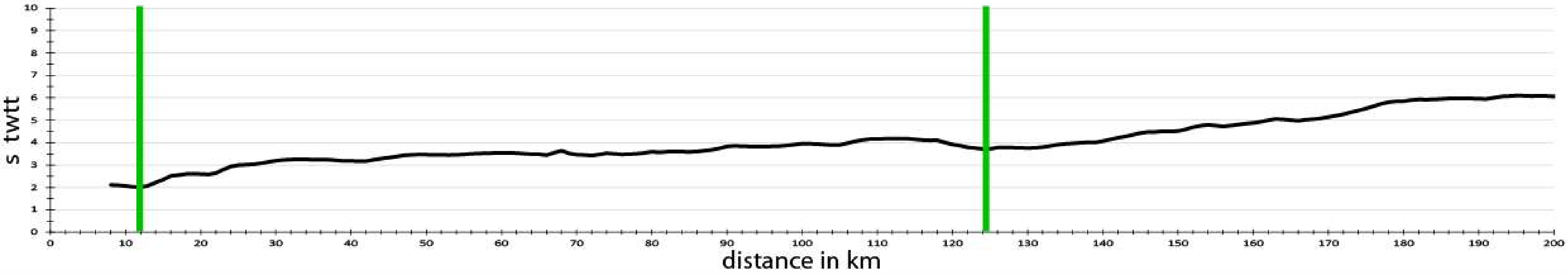

Profile AFR3

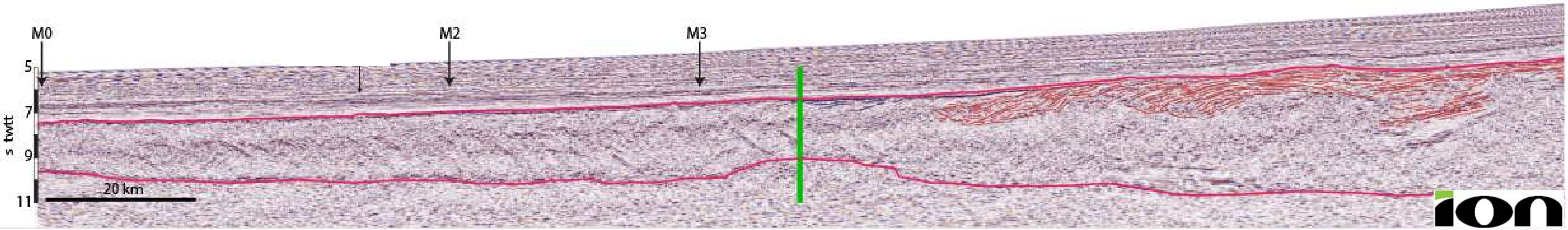

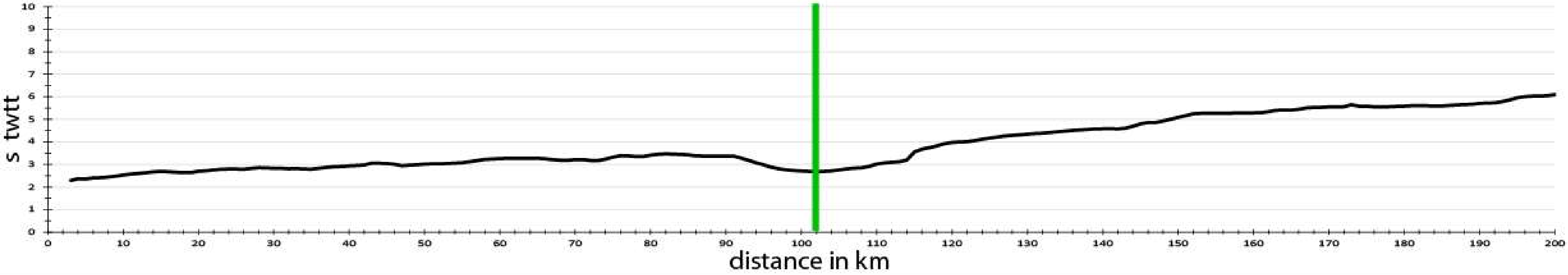

Profile AFR4

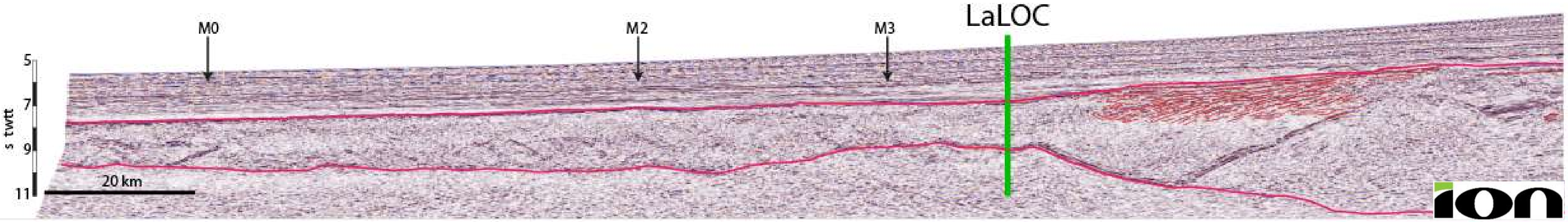

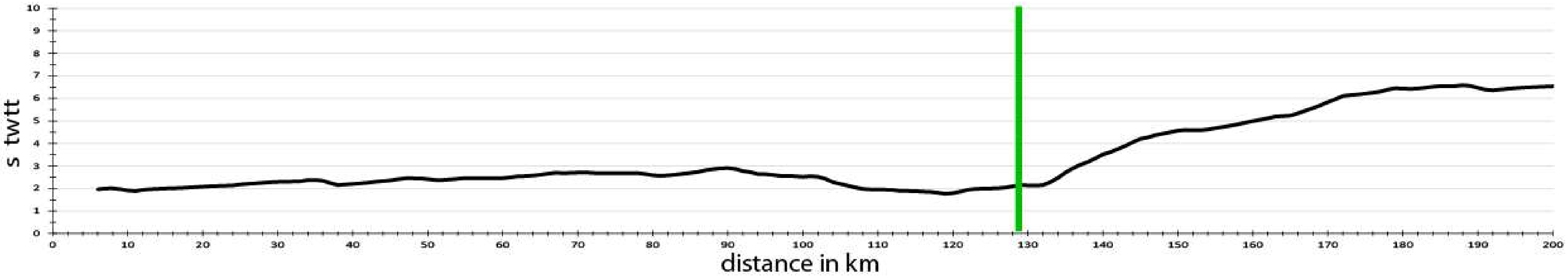

Profile AFR5

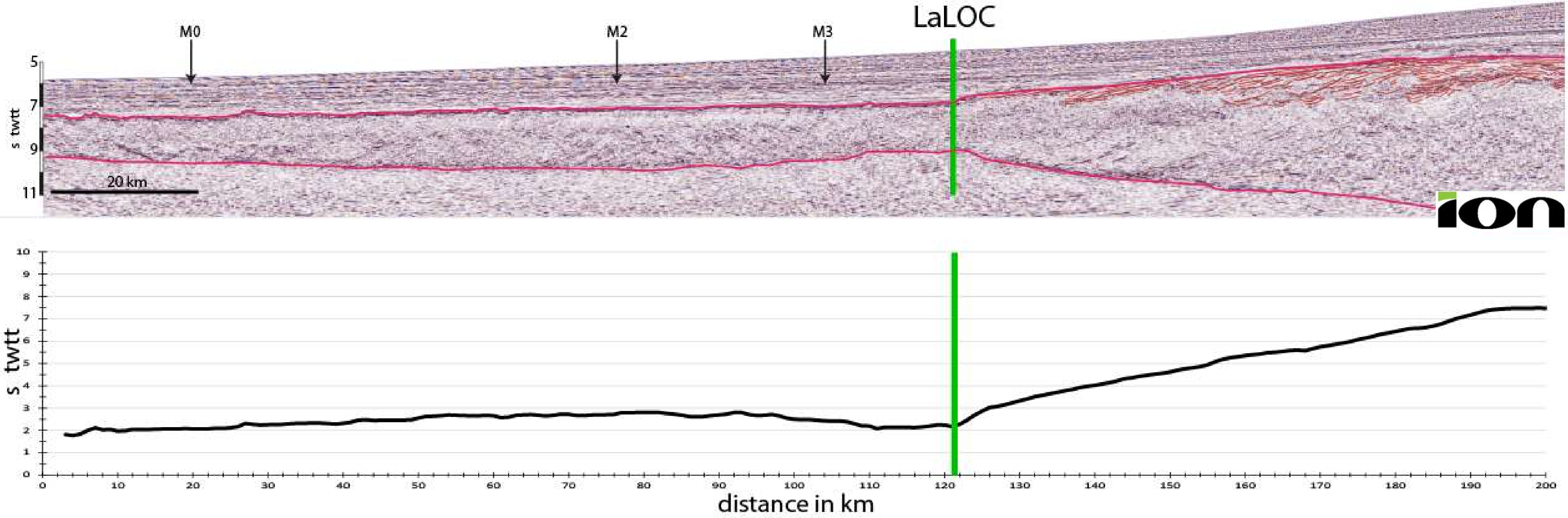

Profile AFR6

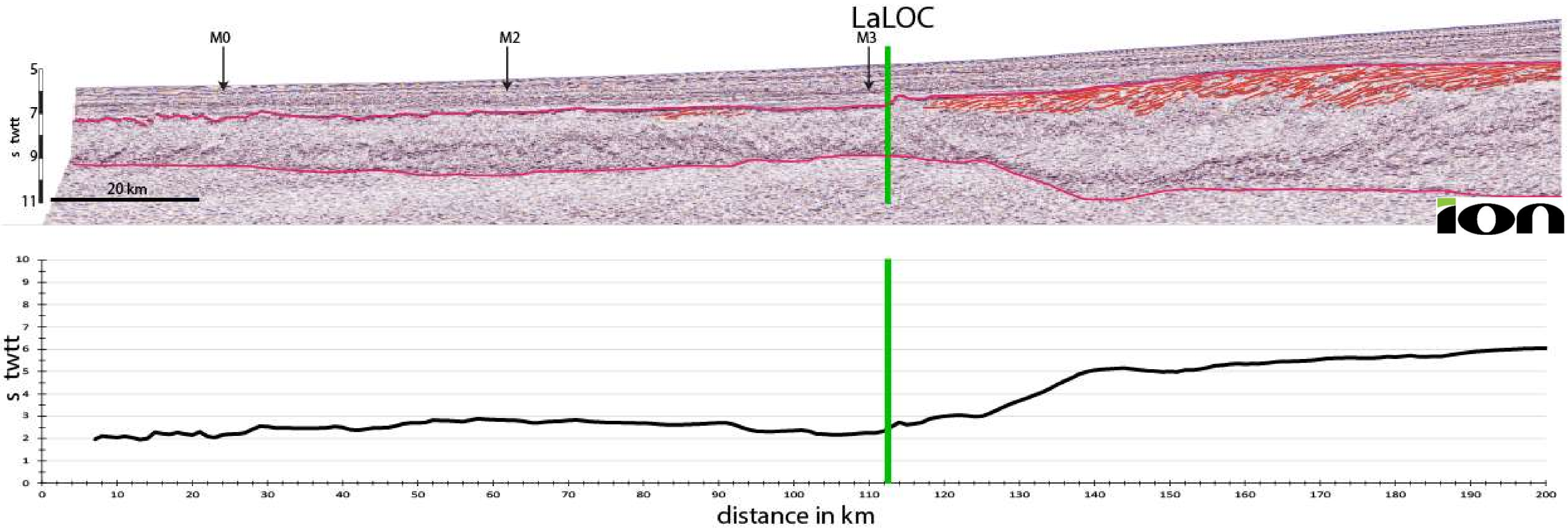

Profile AFR7

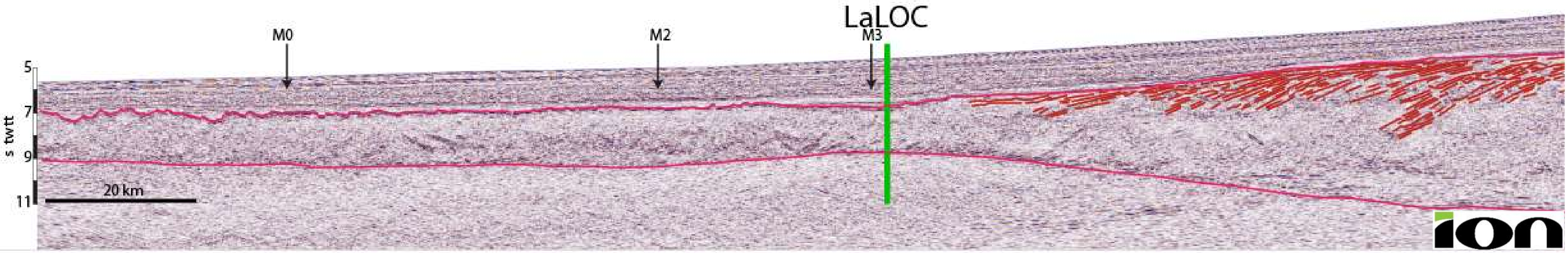

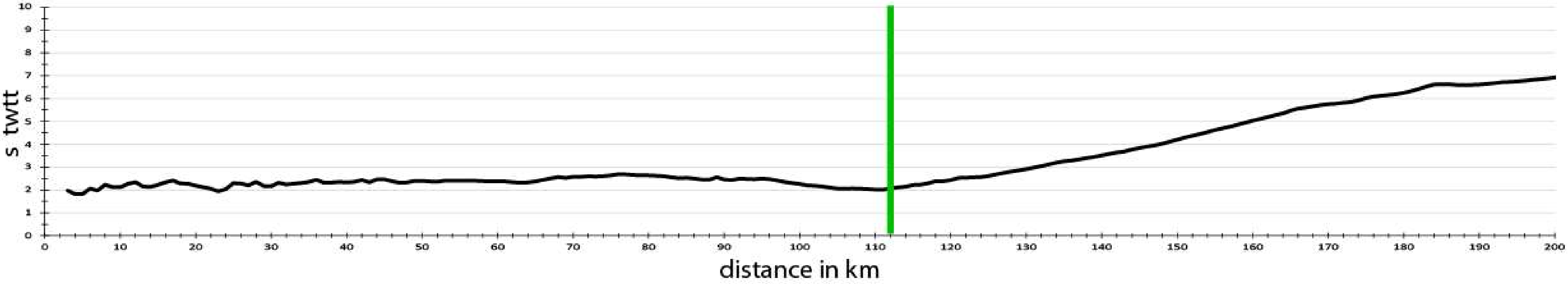

Profile AFR8

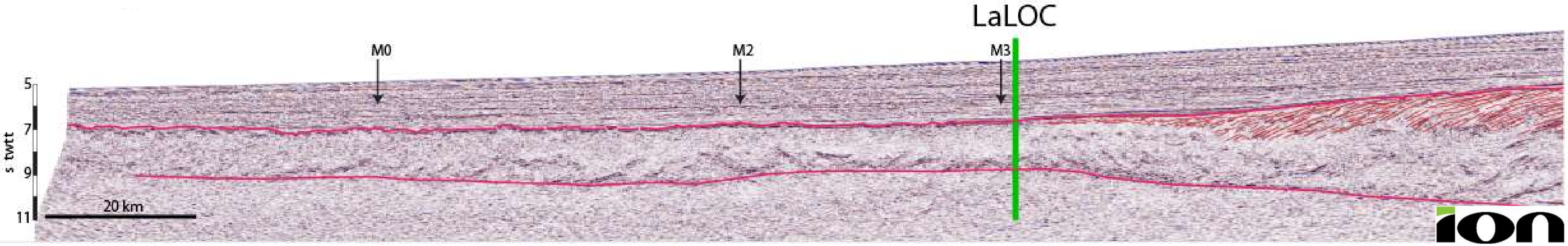

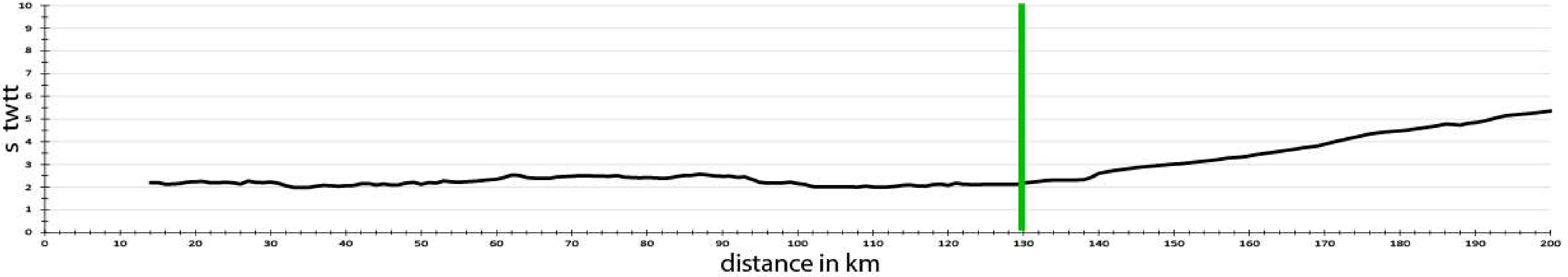

Profile AFR9

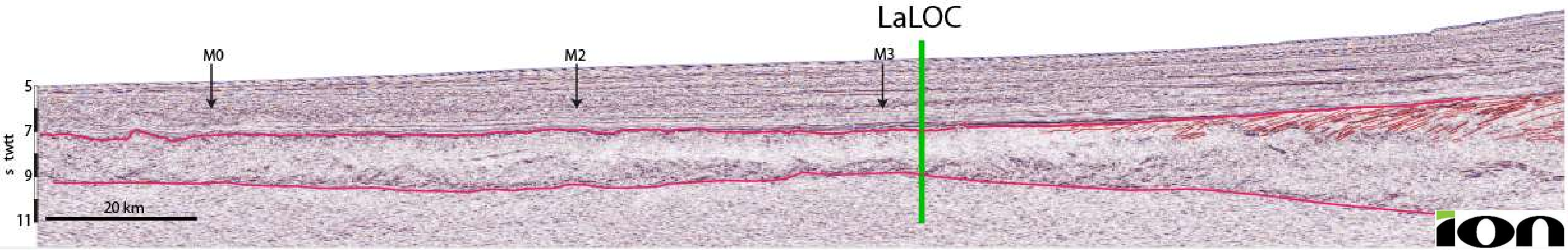

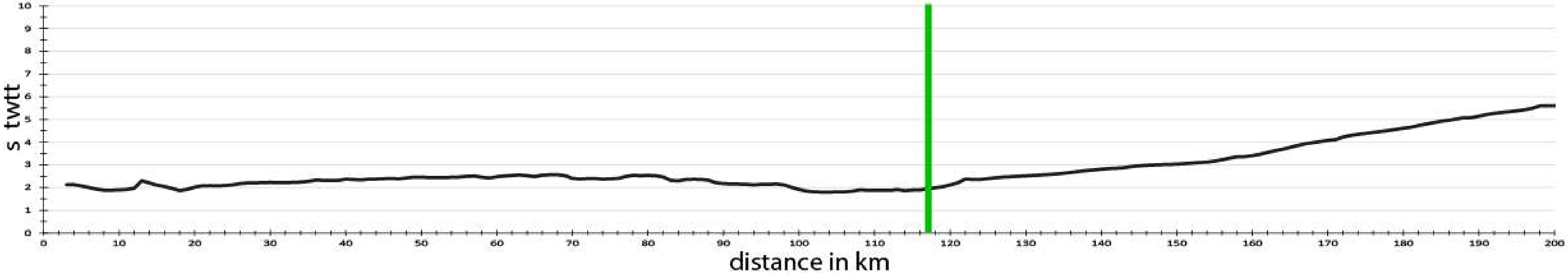

Fig. S3: The next 19 pages show the seismic reflection profiles that we used along the South American margin. Top basement, Moho and SDRs are shown for each profile. The crustal thickness variation is shown below. Green vertical lines are located where top basement shallows while Moho deepens. LaLOC indicates the landward limit of oceanic crust. M4 is for M4.o (127.5 Ma); M3 is for M3.o (126.5 Ma); M2 is M2.o (124.7 Ma) and M0 is for M0.y (121 Ma) and indicate where the profiles cut the isochrones. Courtesy of ION Geophysical & BGR.

Profile SAM1

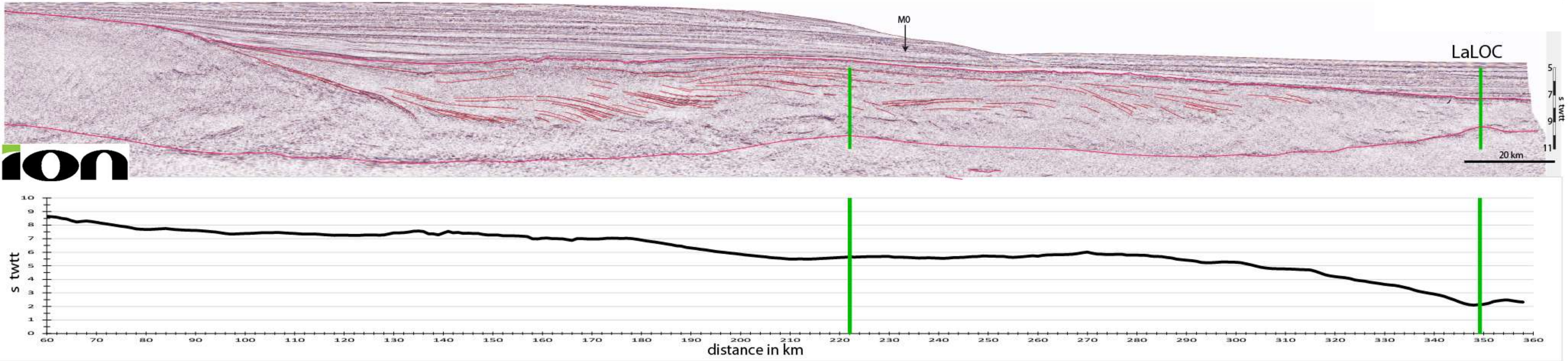

Profile SAM2

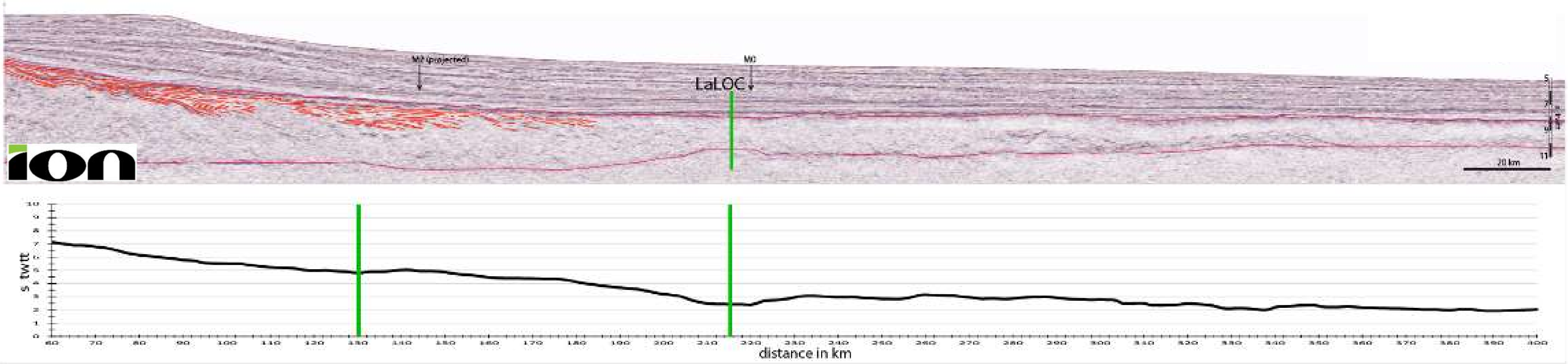

Profile SAM3

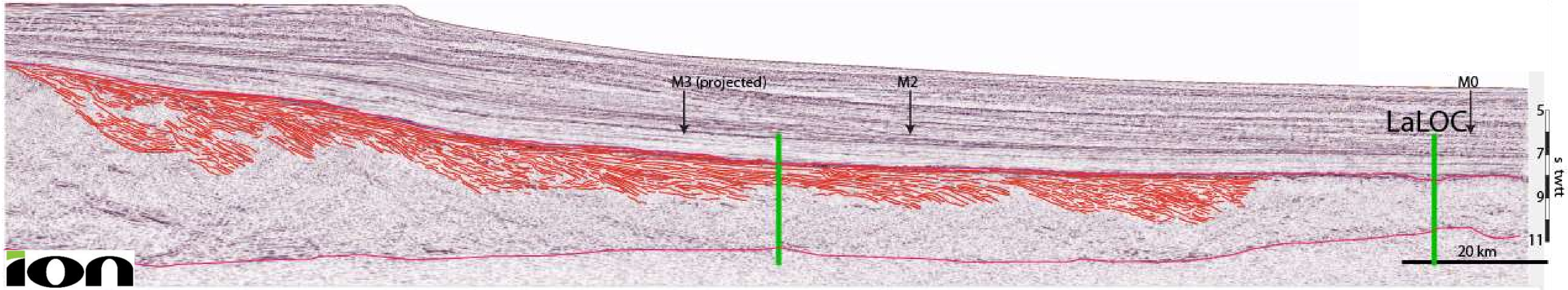

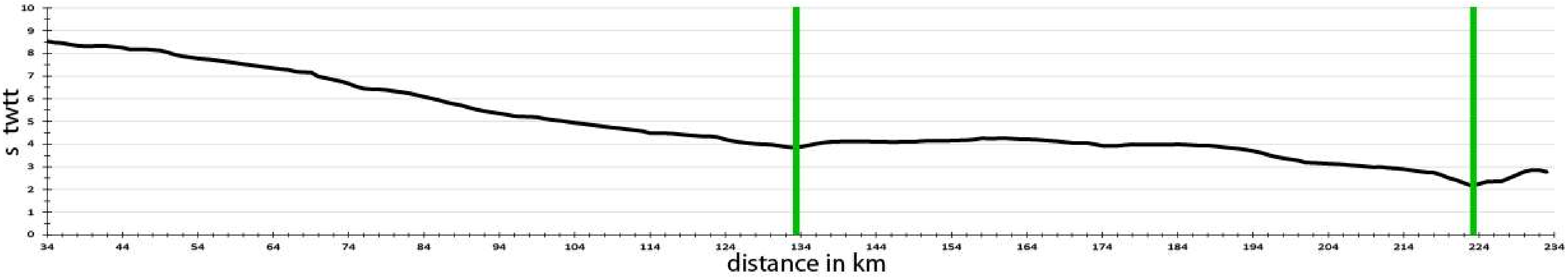

Profile SAM4

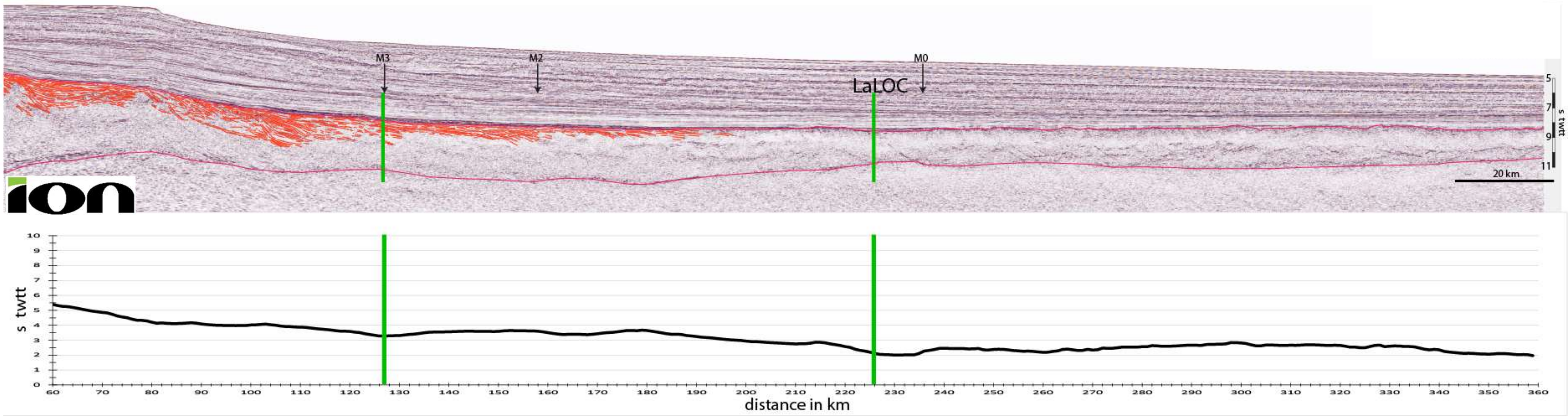

Profile SAM5

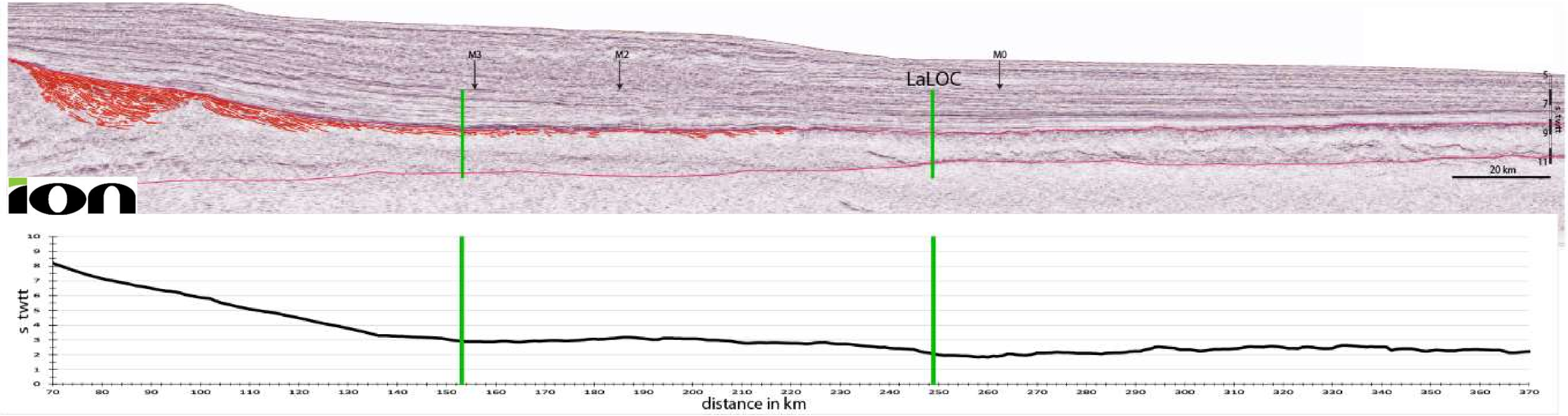

Profile SAM6

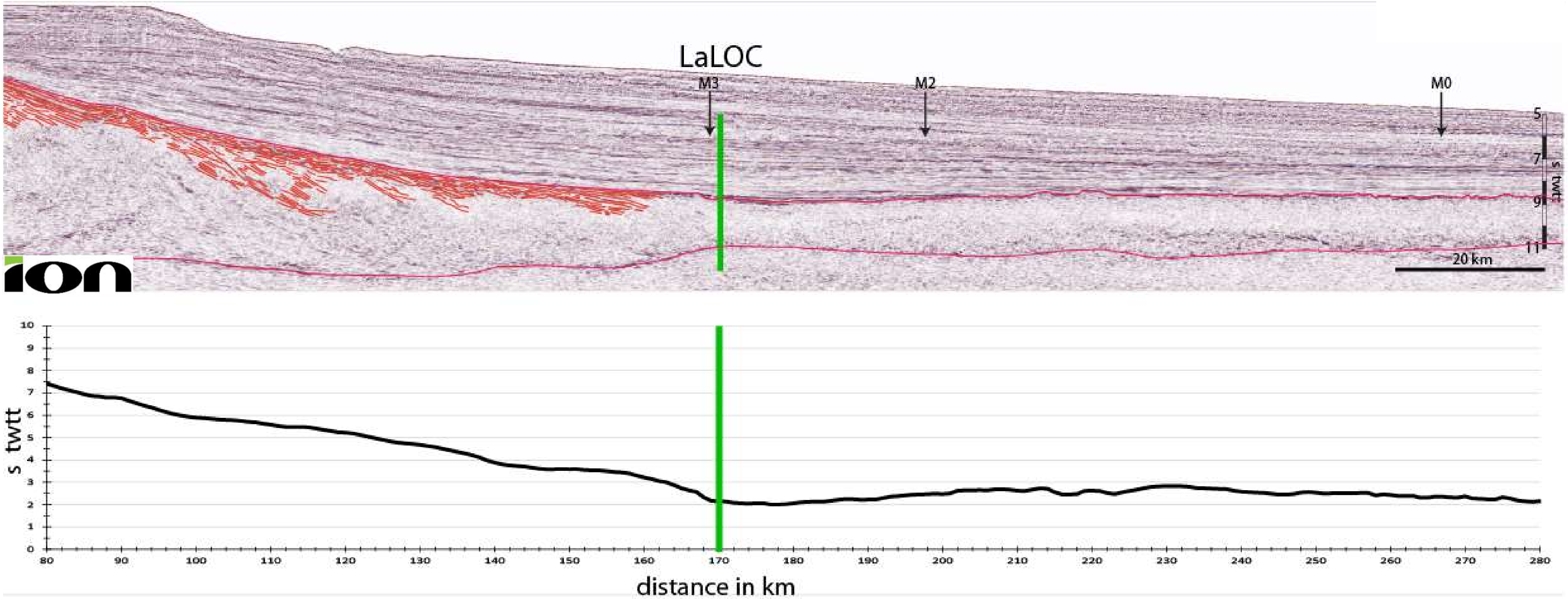

Profile SAM7

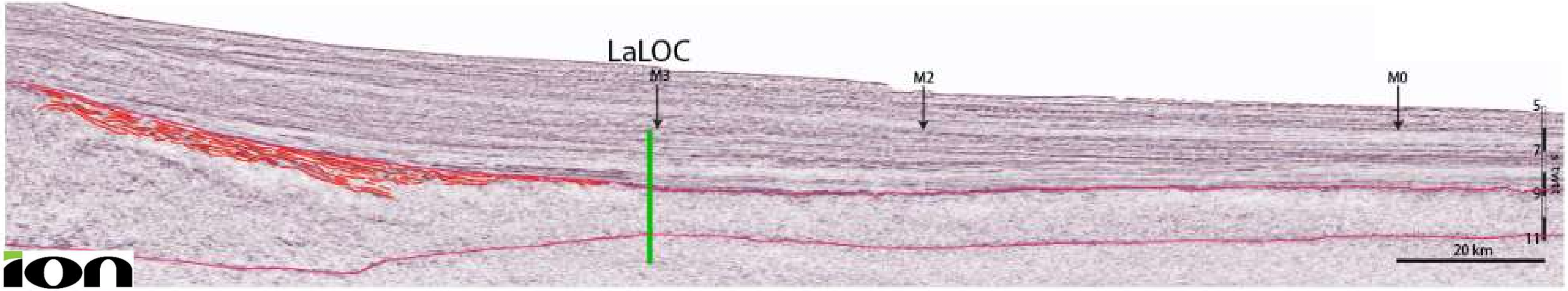

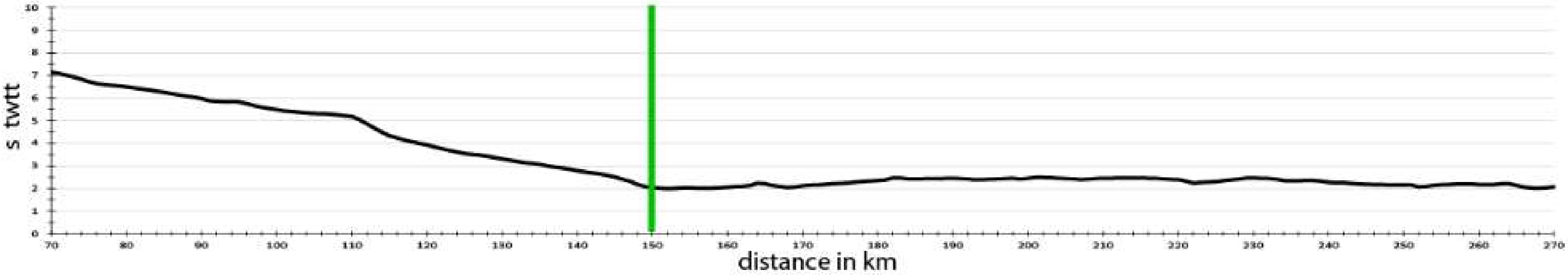

Profile SAM8

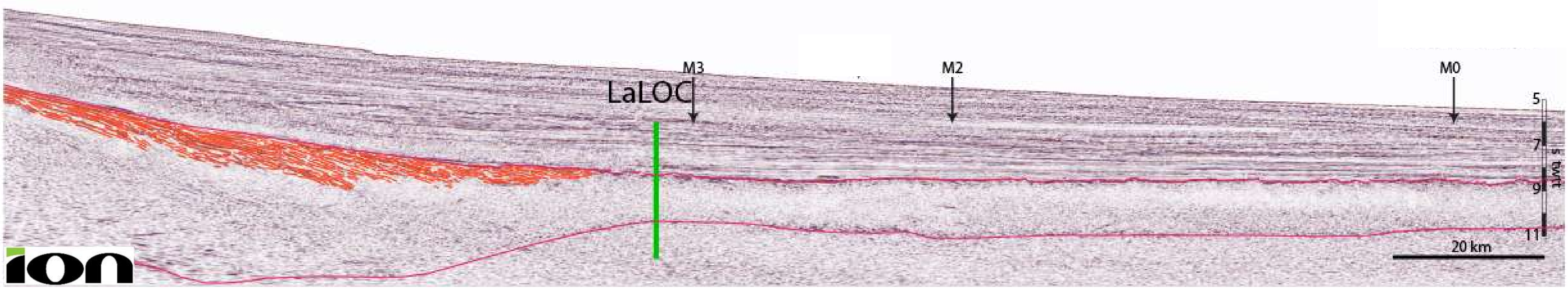

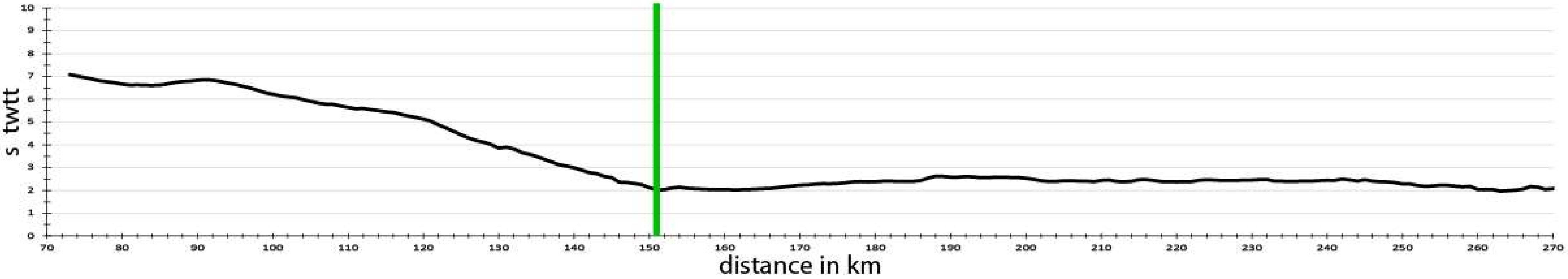

Profile SAM9

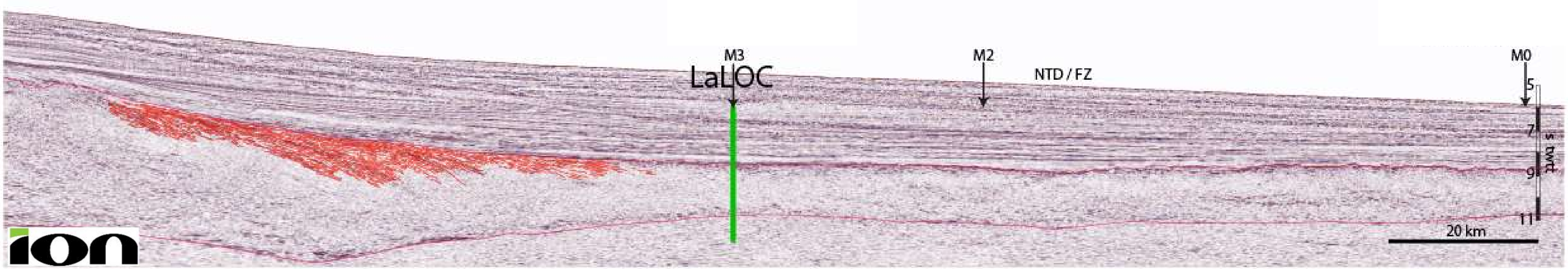

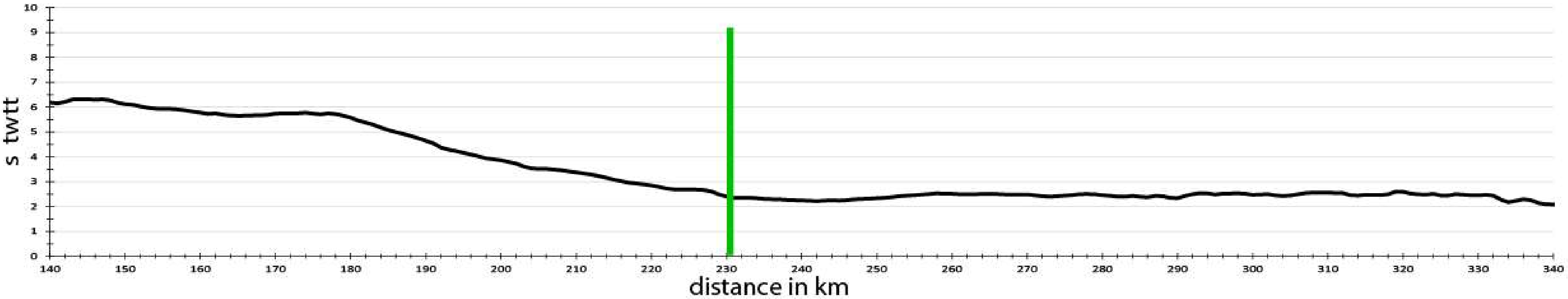

Profile SAM10

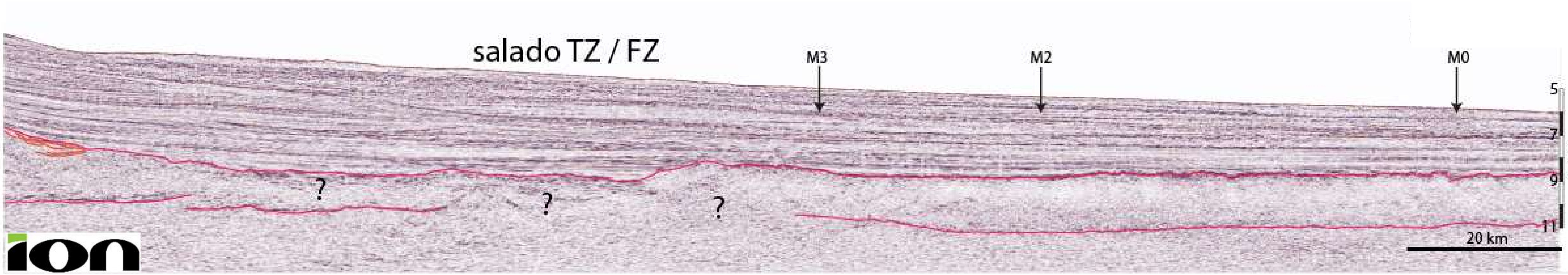

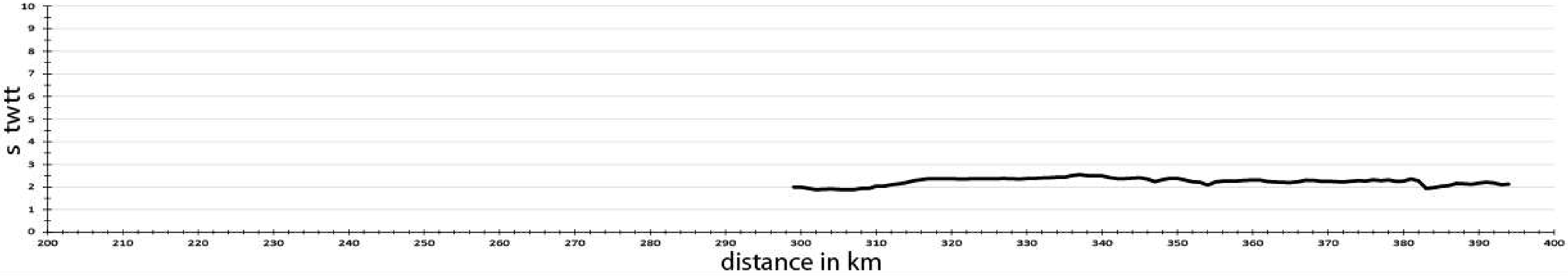

Profile SAM11

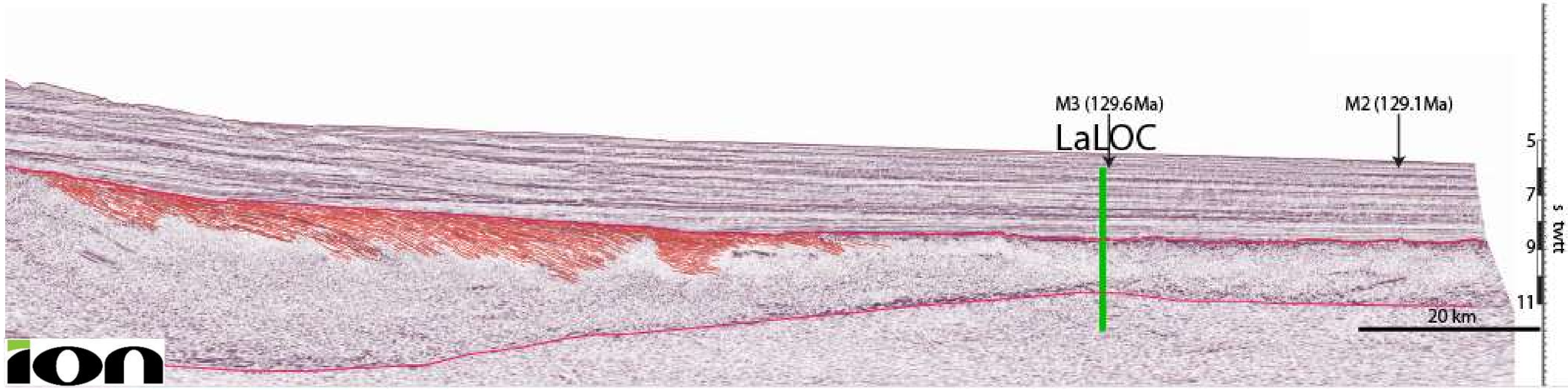

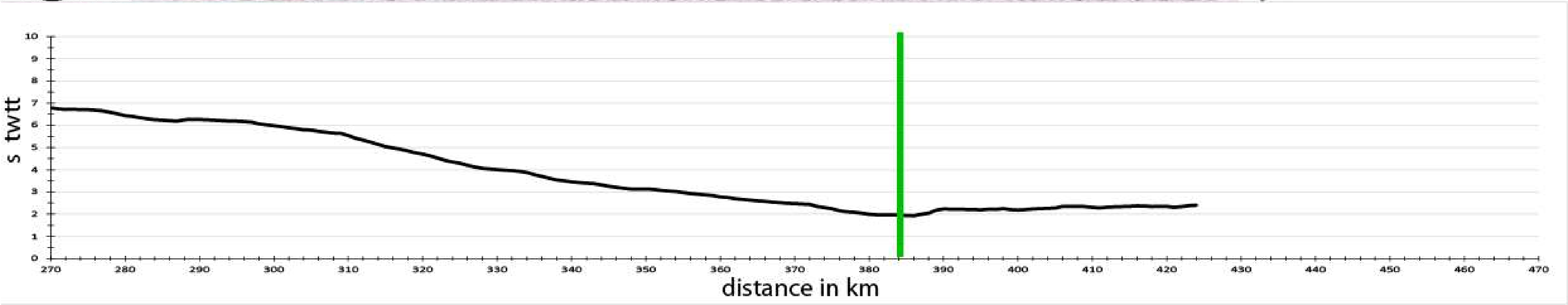

Profile SAM12

copla_02

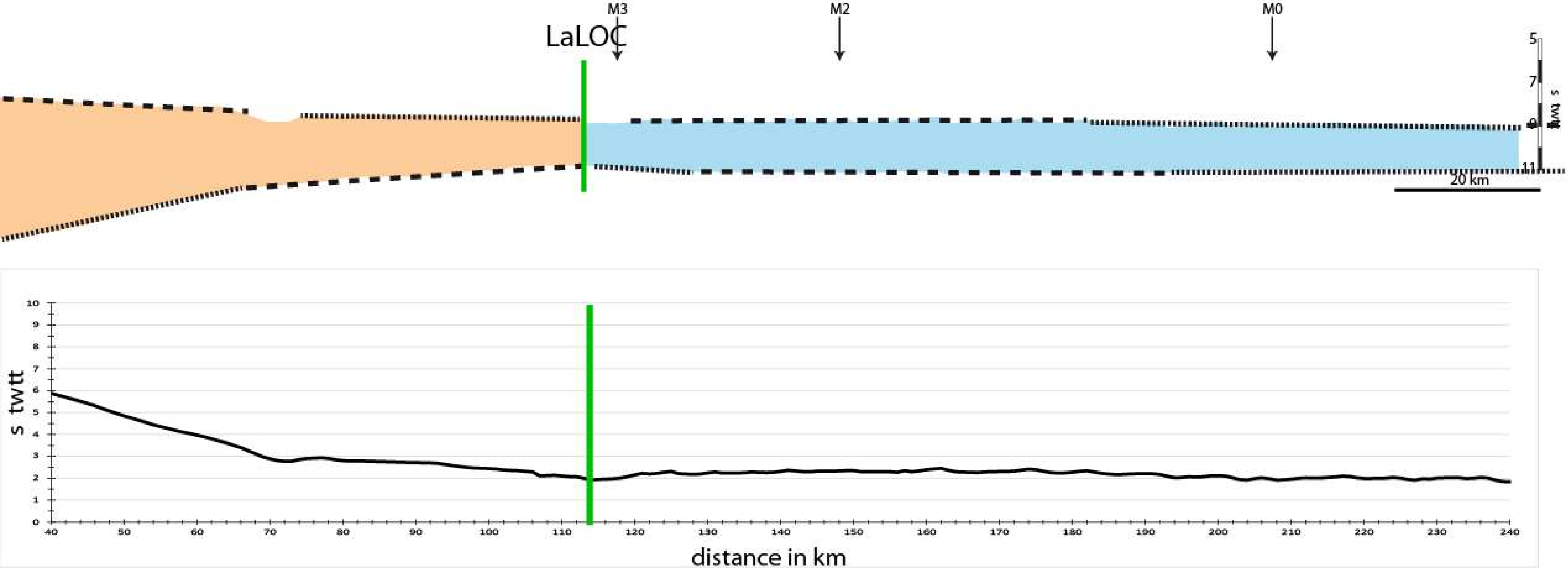

See the copla_02 seismic profile in Soto et al., 2011.

Profile SAM13

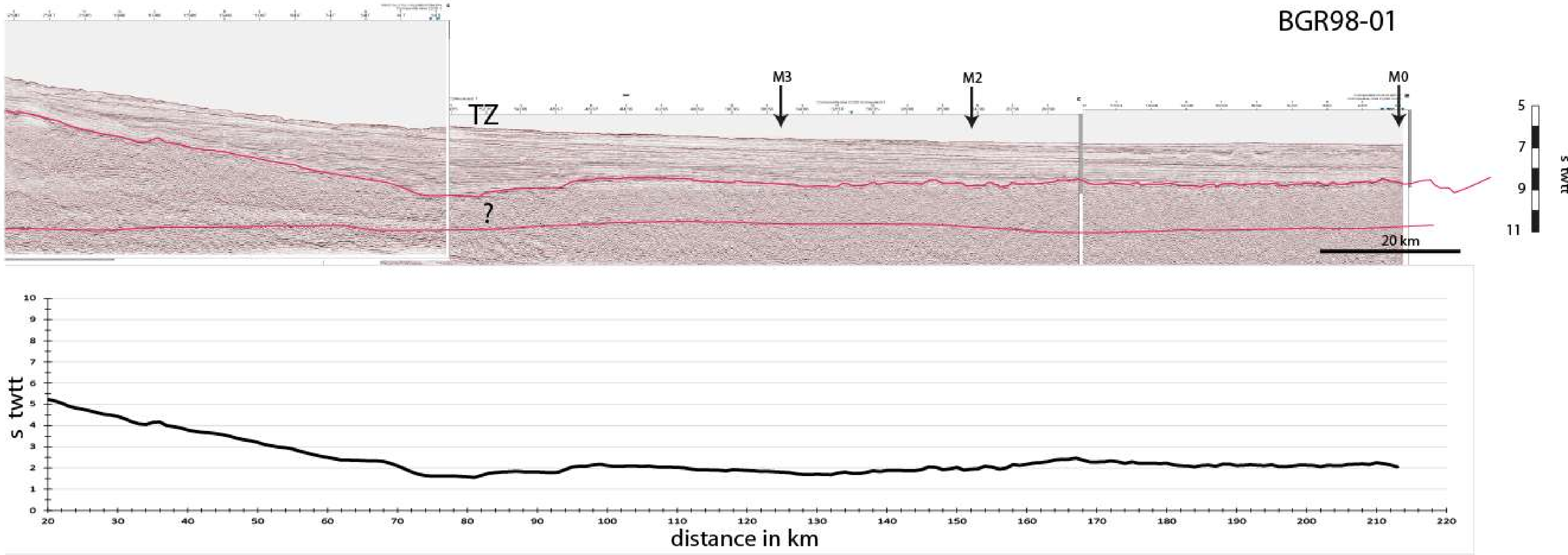

Profile SAM14

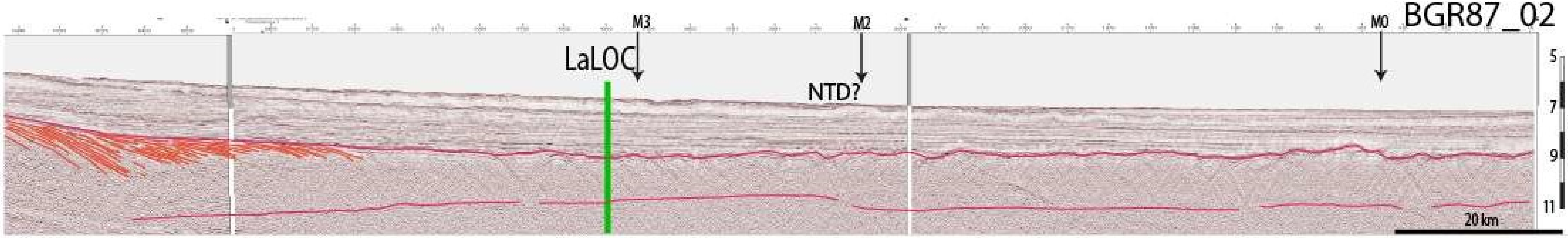

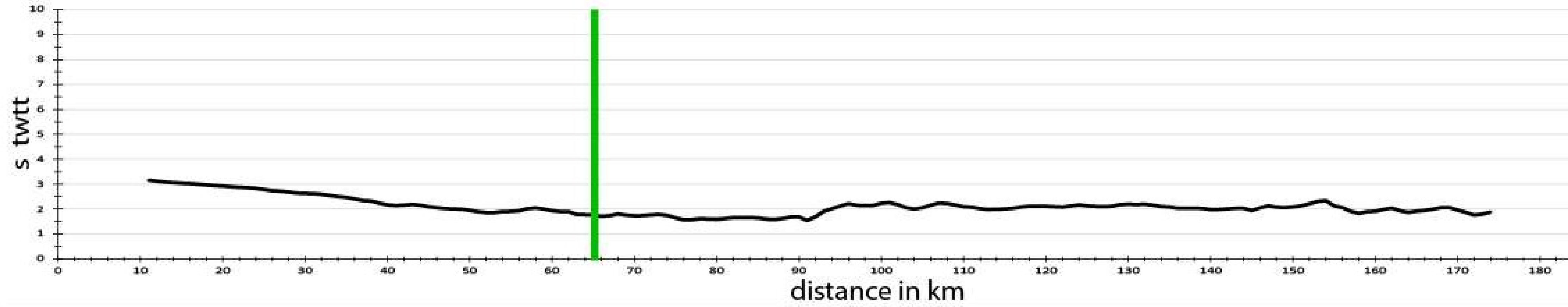

Profile SAM15

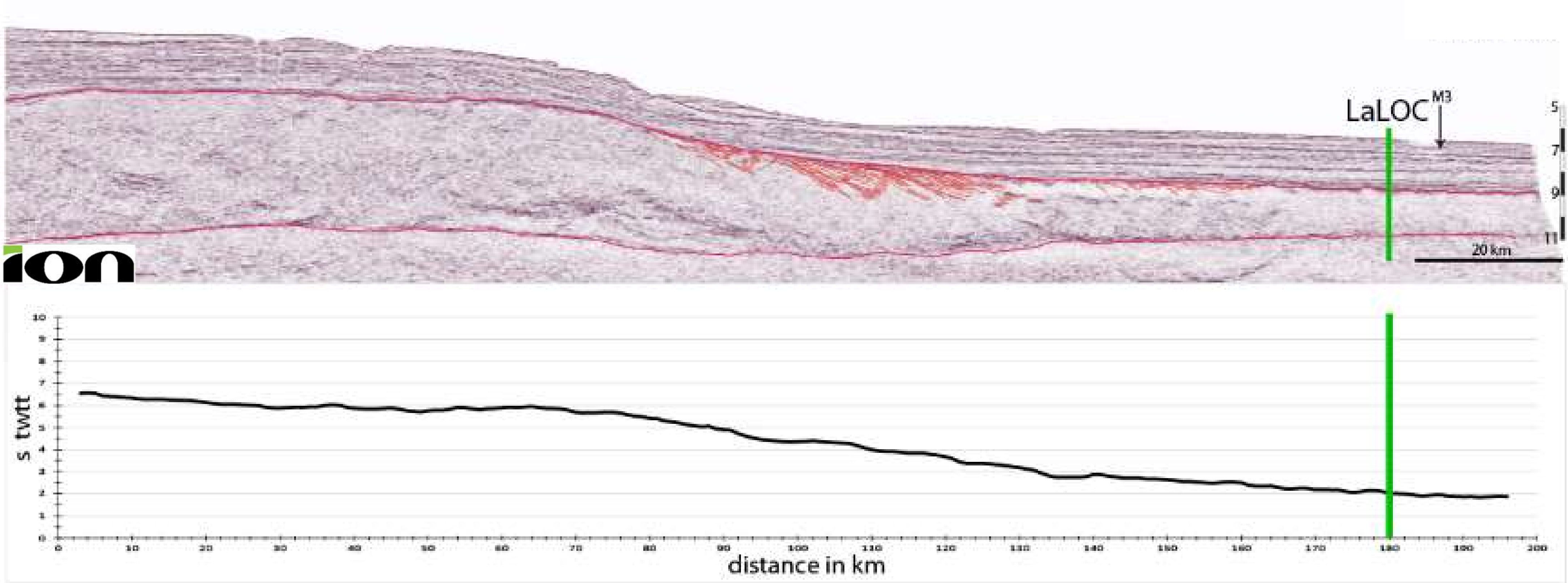

Profile SAM16

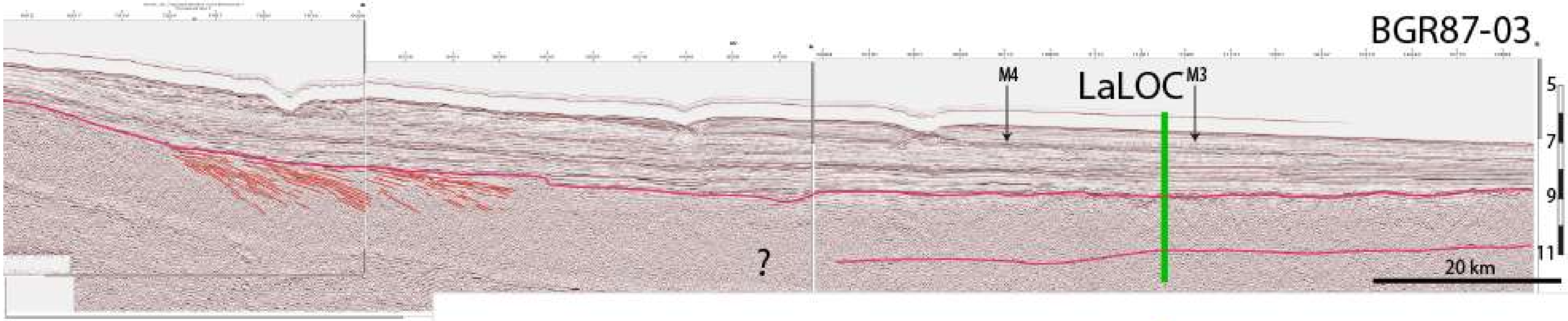

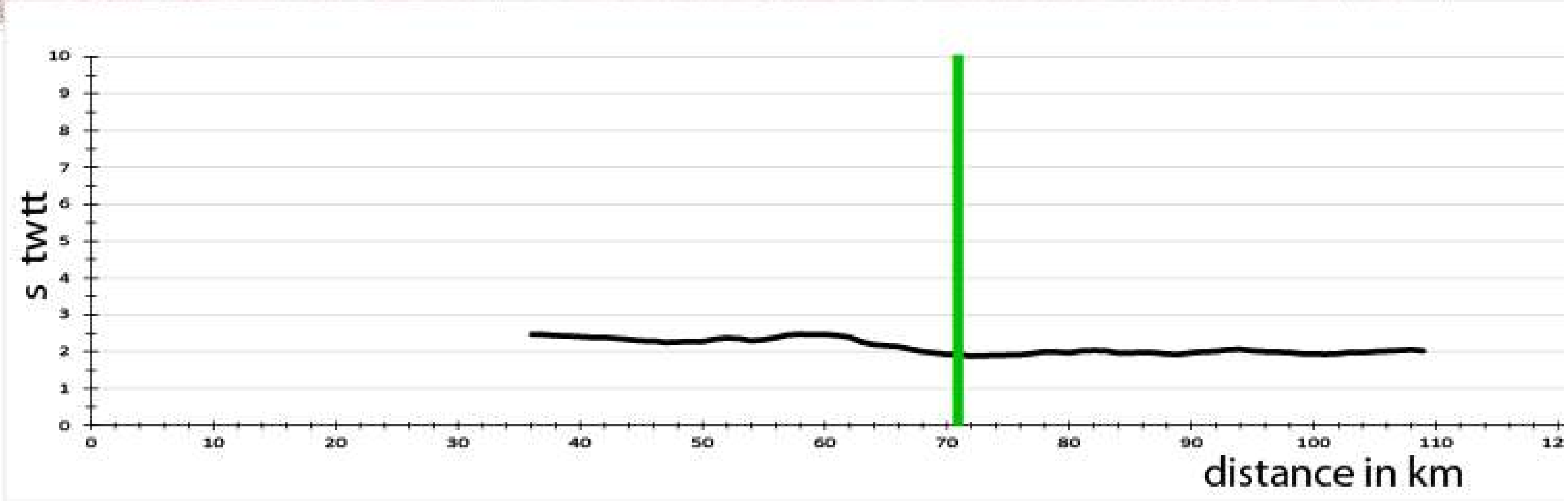

Profile SAM17

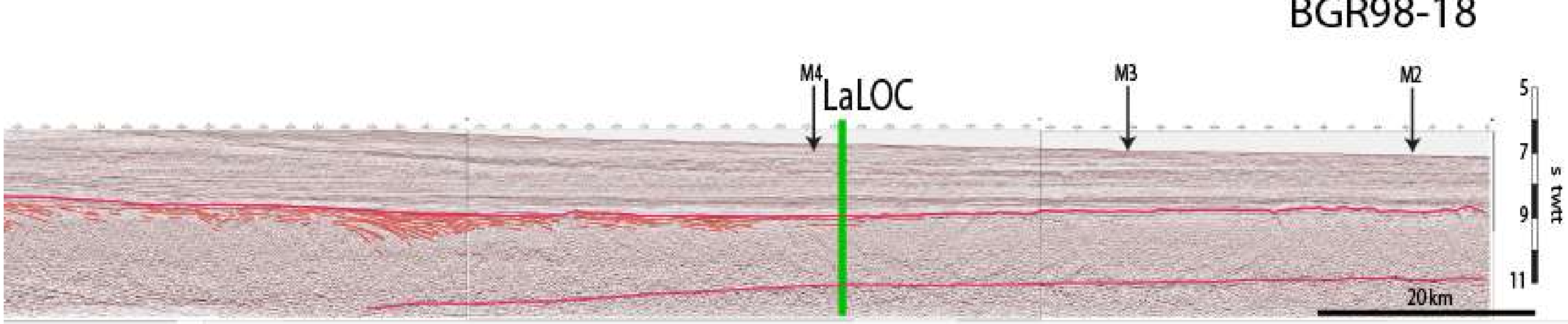

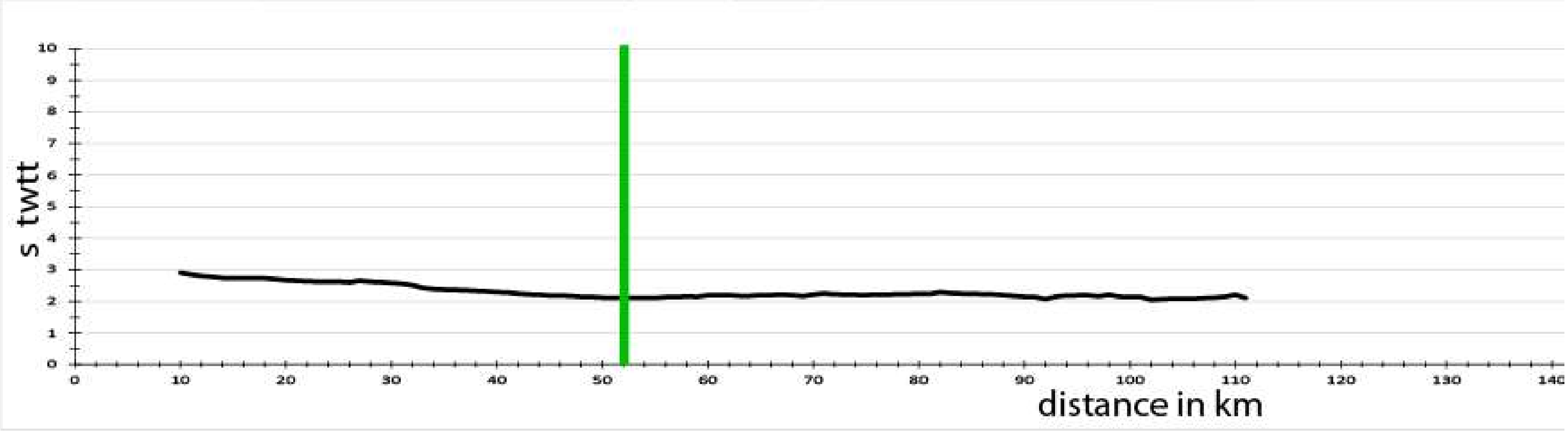

Profile SAM18

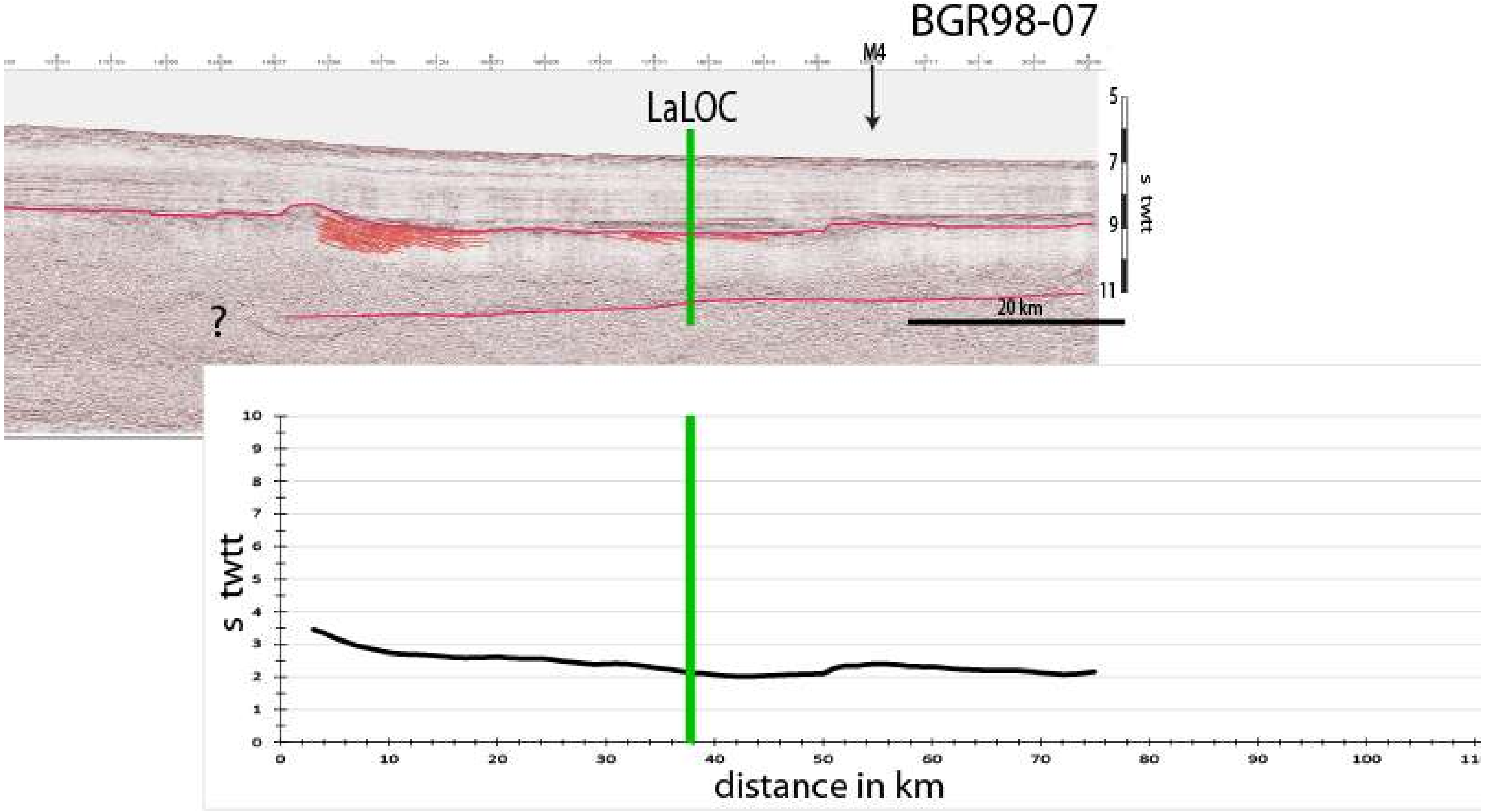

Profile SAM19

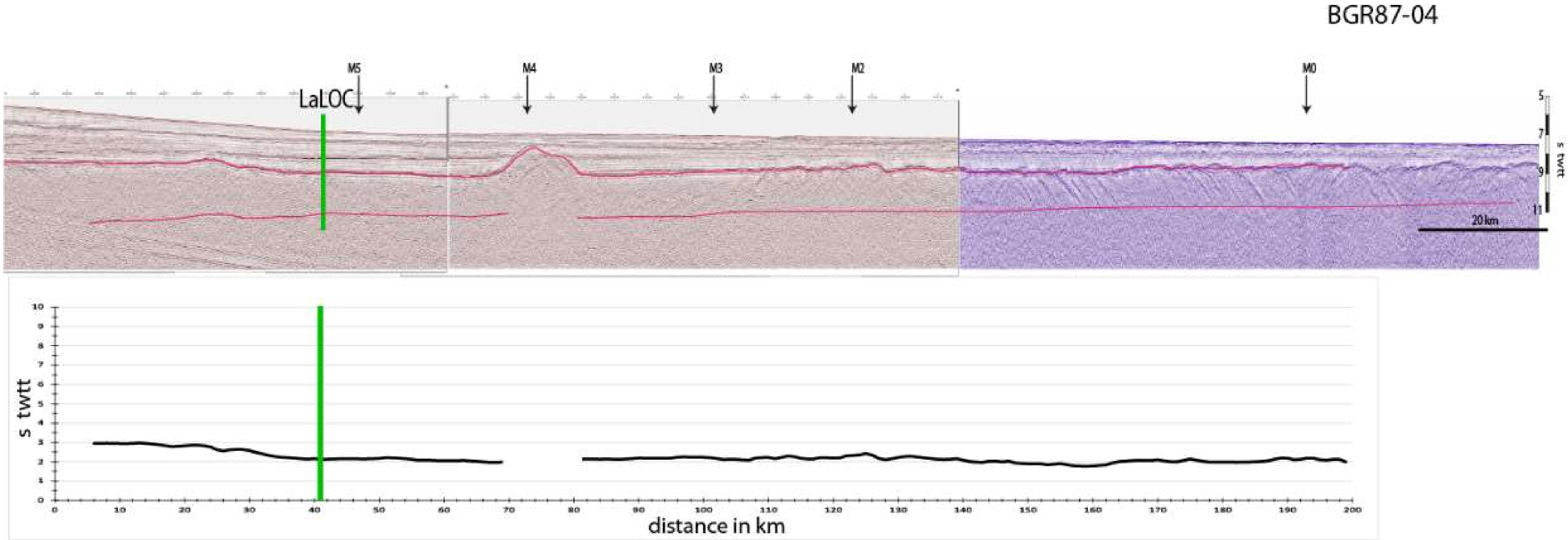

Fig. S4: Comparison between the seismic structure of the oceanic crust observed on the eastern end of profile SAM11 (courtesy of ION Geophysical) and 6 examples at the same scale from the Enderby oceanic basin (after Sauter et al., 2021) (approximately no vertical exaggeration, copyright Commonwealth of Australia - Geoscience Australia).

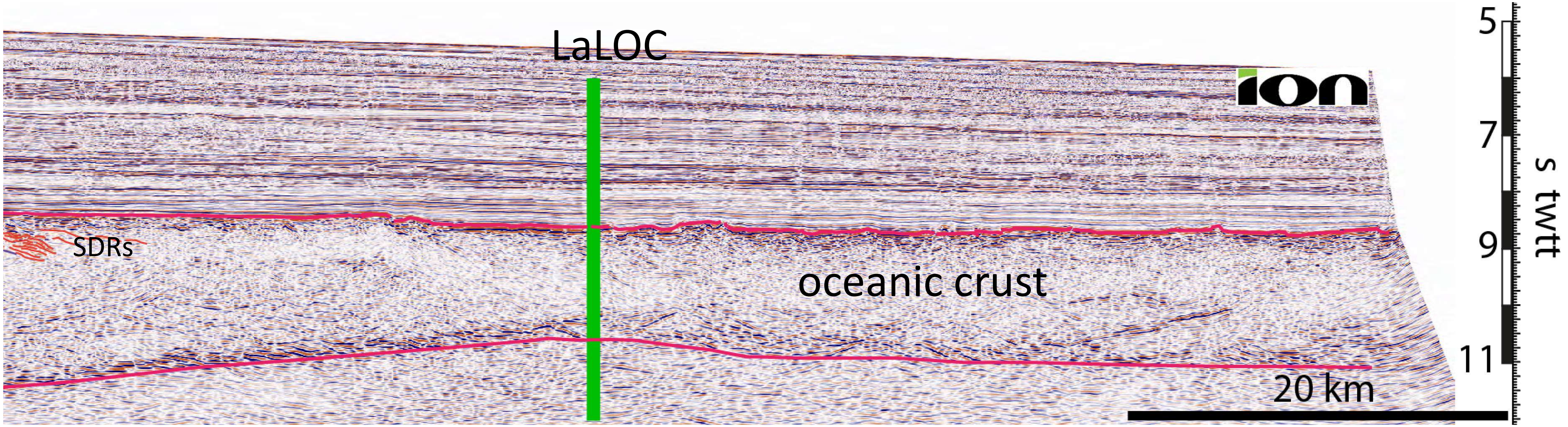

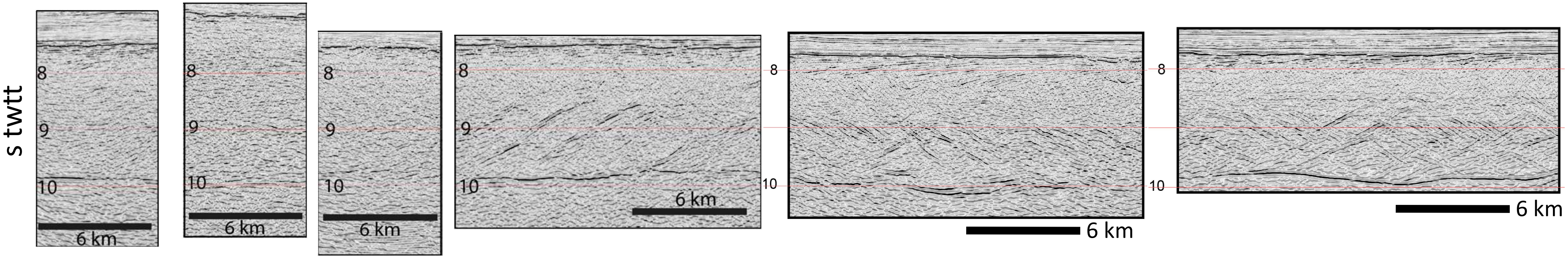

Fig. S5: Compiled isochron map for the South Atlantic using mainly pickings from Collier et al., 2017 completed by those of Granot & Dyment, 2015, Hall et al., 2018 and Koopman et al., 2016. The global Earth Magnetic Anomaly Grid at 2 arc minute resolution version 3 (EMAG2v3) is shown in the background. Blue, red, purple and yellow lines are M0.y, M2.o, M3.o and M4.o isochrones, respectively. The black dashed lines indicating fracture zones are from Chauvet et al., 2020.

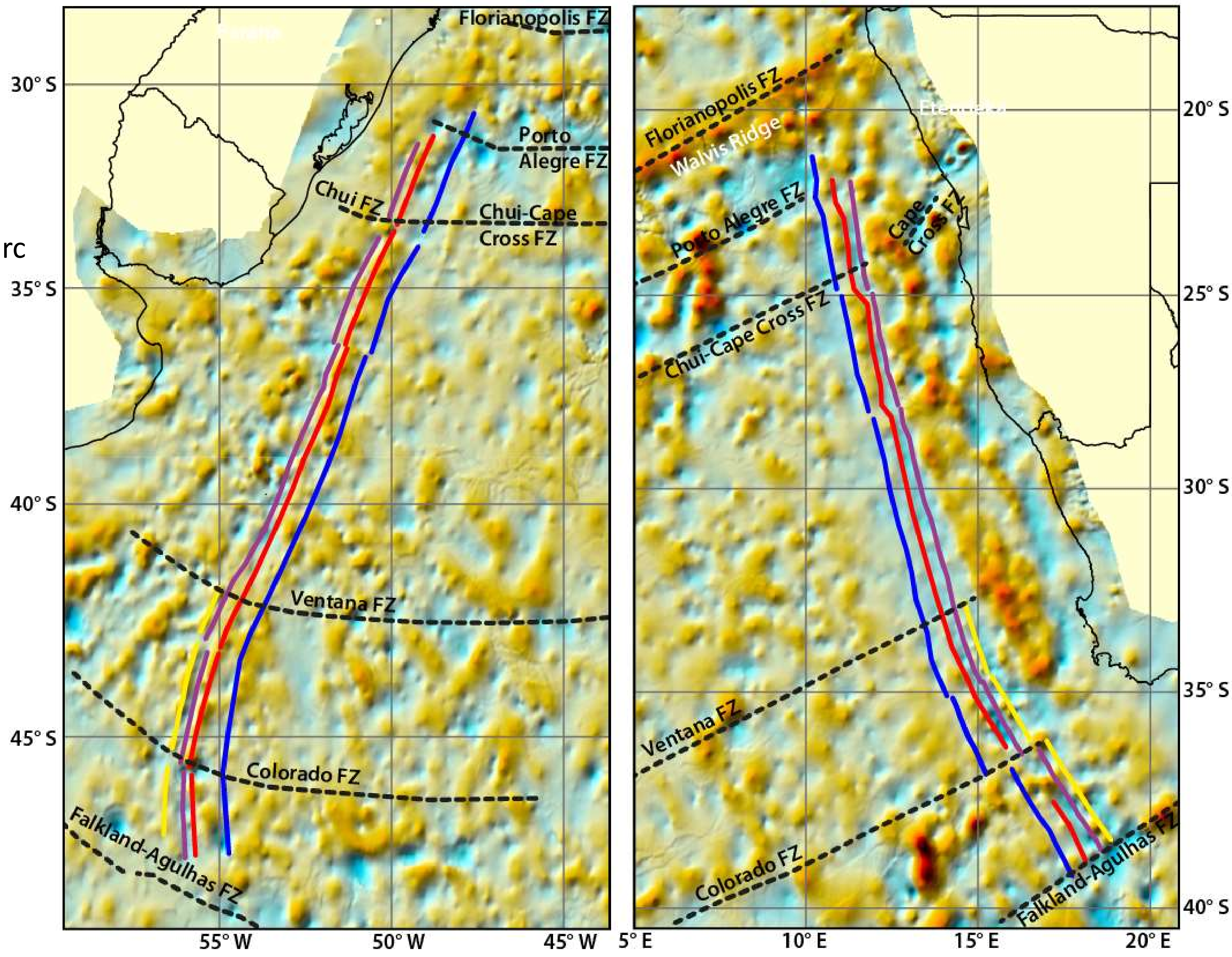

Fig. S6: Comparison between crustal thickness measurements at the LaLOC from Taposeea et al. (2017) and this study. Blue double arrows show the location of the measurements of Taposeea et al. (2017) relative to the LaLOC (in green) as defined in this study for two profiles (SAM7 and SAM17). In profile SAM7 (top) the crustal thickness is taken seaward of the LaLOC while in profile SAM17 (bottom) the crustal thickness is taken landward of the LaLOC (in the outer SDRS domain) by Taposeea et al. (2017). In both cases the crust measured by Taposeea et al. (2017) is thicker than the crust at the LaLOC as defined in this study (2.55 and 2,23 s TWTT for SAM7 and SAM17 relative to 2.01 and 2.11 s TWTT for this study).

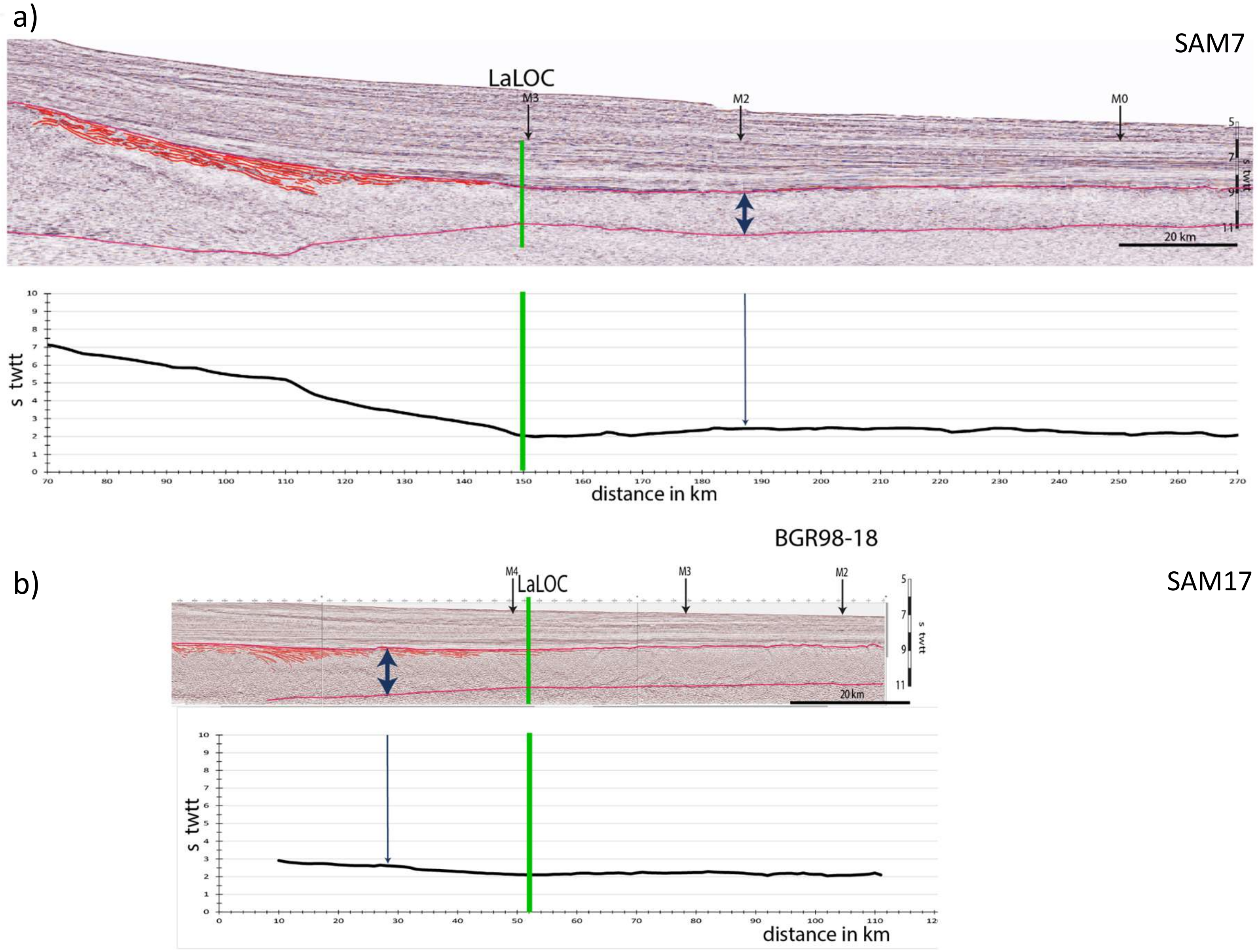

Fig. S7: Variation of the crustal thickness north of Chui FZ along 5 seismic reflection profiles across the South American margin and 4 profiles across the West African margin. The origin of the x-axis is located where the top basement and Moho diverge landward of the LaLOC (see Figs. 2 & S2-S3). Black squares and circle indicate M0 and M3 locations, respectively, for each profile. The dashed lines indicate the oceanic crustal variation along the northern seismic profile south of the Chui FZ for comparison. Thickness of the lines increases northward. Red lines show the overall trends of the crustal thickness variations.

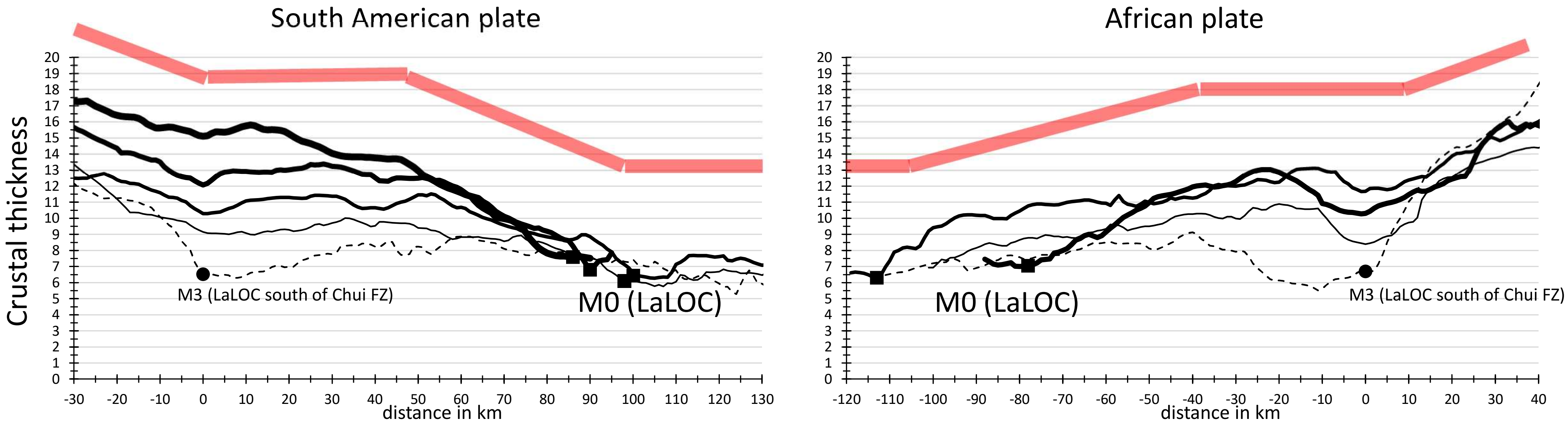

Fig. S8: Variation of the crustal thickness along one strike seismic line located close to the M3.o isochron alongside the Namibian margin Courtesy of ION Geophysical.

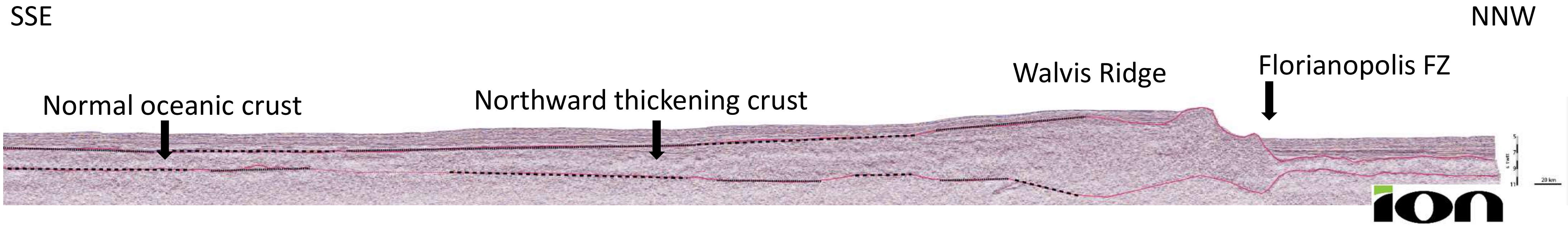

Fig. S9: Crustal two way travel time versus crustal thickness. We converted TWTT in kilometres using 6.4 km/s bulk velocity obtained by Hoggard et al. (2017) for more than ~30 Ma old oceanic crust (red line). The obtained crustal thicknesses are close to the ones obtained using the relationship of Canales et al. (2003) : y [km] = 3.054x [s TWTT] + 0.261 (blue line) for magmatic oceanic crust at the fast spreading East Pacific Rise. The mean difference between the two estimations is 0.03 km for 1-3 s TWTT. For comparison the black line shows thicknesses convert with the 6.7 km/s bulk velocity from White et al. (1992).

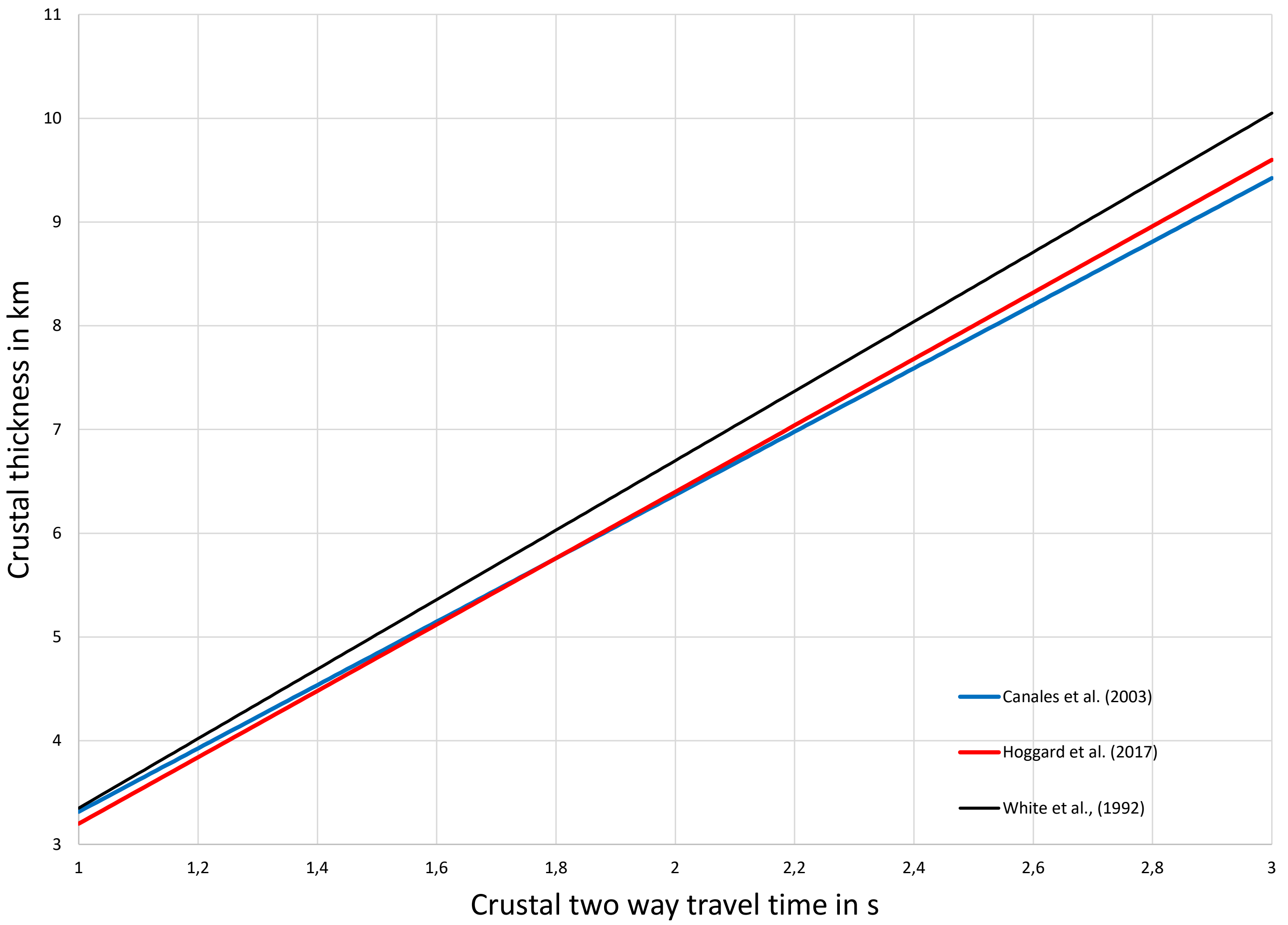

Table S1: Magnetic anomalies used in this study. Ages are from Ogg 2020

**Magnetic anomalies**	**Age (Ma) Ogg (2020)**
M9r.o	130.297
M4.o	127.526
M3.o	126.514
M2.o	124.717
M0.y	120.964

Table S2: Mean crustal thickness measured at LaLOC. We converted TWTT in kilometres using 6.4 km/s bulk velocity obtained by Hoggard et al. (2017) for more than ~30 Ma old oceanic crust (see Fig. S9).

**SOUTH AMERICA**					**AFRICA**			
**Profile Name**	**Crustal thickness at LaLOC in s TWTT (5 km average within the oceanic crust)**	**in km**	**Age**	**comment**	**Profile Name**	**Crustal thickness at LaLOC in s TWTT (5 km average within the oceanic crust)**	**in km**	**Age**
				**COLORADO FZ**				
SAM19 BGR87-04	2,14	6,85	>M4					
SAM18 BGR98-07	2,04	6,53	>M4					
SAM17 BGR98-18	2,11	6,75	~M4					
SAM16 BGR87-03	1,89	6,05	M3					
				**VENTANA FZ**				
SAM15	1,97	6,30	M3					
SAM14 BGR87-02	1,75	5,60	M3					
SAM13 BGR98-01				crosses a FZ				
SAM12 copla-02	1,96	6,27	M3	Soto et al., 2011	AFR9	1,9	6,08	M3
SAM11	1,97	6,30	M3		AFR8	2,12	6,78	M3
SAM10				crosses a FZ	AFR7	2,04	6,53	M3
				**RIO DE LA PLATA FZ**				
SAM9	2,3	7,36	M3		AFR6	2,23	7,14	M3
SAM8	2,08	6,66	M3					
SAM7	2,01	6,43	M3		AFR5	2,15	6,88	M3
SAM6	2,06	6,59	M3		AFR4	2,03	6,50	M3
				**CHUI FZ**				
SAM5	1,94	6,21	M0					
SAM4	2,04	6,53	M0		AFR2	2,1	6,72	M0
SAM3	2,29	7,33	M0					
SAM2	2,43	7,78	M0		AFR1	2,24	7,17	M0
				**PORTO ALEGRE FZ**				
SAM1	2,15	6,88	~116 Ma	within the CQZ				
				**FLORIANOPOLIS FZ**				
Mean SAM	2,07	6,61			Mean AFR	2,1	6,72	
Std SAM	0,16	0,53			Std AFR	0,11	0,35	
Mean SAM+AFR	2,08	6,65						
Std SAM+AFR	0,15	0,47						

References used in the supplementary file:

- Canales, J.P., Detrick, R.S., Toomey, D.R., and Wilcock, S.D., 2003, Segment-scale variations in crustal structure of 150- to 300-k.y.-Old fast spreading oceanic crust (East Pacific Rise, 8°15'N-10°15'N from wide-angle seismic refraction profiles: Geophysical Journal International, v. 152, p. 766-794.
- Chauvet, F., Sapin, F., Geoffroy, L., Ringenbach, J.-C., and Ferry, J.-N., 2020, Conjugate volcanic passive margins in the austral segment of the South Atlantic – Architecture and development: Earth-Science Reviews, p. 103461, doi:https://doi.org/10.1016/j.earscirev.2020.103461.
- Collier, J.S., McDermott, C., Warner, G., Gyori, N., Schnabel, M., McDermott, K., and Horn, B.W., 2017, New constraints on the age and style of continental breakup in the South Atlantic from magnetic anomaly data: Earth and Planetary Science Letters, v. 477, p. 27-40, doi:https://doi.org/10.1016/j.epsl.2017.08.007.
- Granot, R., and Dyment, J., 2015, The Cretaceous opening of the South Atlantic Ocean: Earth and Planetary Science Letters, v. 414, p. 156-163, http://dx.doi.org/10.1016/j.epsl.2015.01.015.
- Hall, S.A., Bird, D.E., McLean, D.J., Towle, P.J., Grant, J.V., and Danque, H.A., 2018, New constraints on the age of the opening of the South Atlantic basin: Marine and Petroleum Geology, doi:https://doi.org/10.1016/j.marpetgeo.2018.03.010.
- Hoggard, M.J., Winterbourne, J., Czarnota, K., and White, N., 2017, Oceanic residual depth measurements, the plate cooling model, and global dynamic topography: Journal of Geophysical Research: Solid Earth, v. 122, p. 2328– 2372, , doi:10.1002/2016jb013457.
- Koopmann, H., Schreckenberger, B., Franke, D., Becker, K., and Schnabel, M., 2016, The late rifting phase and continental break-up of the southern South Atlantic: the mode and timing of volcanic rifting and formation of earliest oceanic crust: Geological Society, London, Special Publications, v. 420, p. 315-340, doi:10.1144/sp420.2.
- Ogg, J.G., 2020, Chapter 5 - Geomagnetic Polarity Time Scale, *in* Gradstein, F.M., Ogg, J.G., Schmitz, M.D., and Ogg, G.M., eds., Geologic Time Scale 2020, Elsevier, p. 159-192, doi:https://doi.org/10.1016/B978-0-12-824360-2.00005-X.
- Sauter, D., Werner, P., Ceuleneer, G., Manatschal, G., Rospabé, M., Tugend, J., Gillard, M., Autin, J., and Ulrich, M., 2021, Sub-axial deformation in oceanic lower crust: Insights from seismic reflection profiles in the Enderby Basin and comparison with the Oman ophiolite: Earth and Planetary Science Letters, v. 554, p. 116698, doi:https://doi.org/10.1016/j.epsl.2020.116698.
- Soto, M., Morales, E., Veroslavsky, G., de Santa Ana, H., Ucha, N., and Rodríguez, P., 2011, The continental margin of Uruguay: Crustal architecture and segmentation: Marine and Petroleum Geology, v. 28, p. 1676-1689, doi:https://doi.org/10.1016/j.marpetgeo.2011.07.001
- Taposeea, C. A., Armitage, J. J. & Collier, J. S., 2017, Asthenosphere and lithosphere structure controls on early onset oceanic crust production in the southern South Atlantic. Tectonophys. 716, 4-20, doi:https://doi.org/10.1016/j.tecto.2016.06.026
- White, R.S., McKenzie, D., and O'Nions, K., 1992, Oceanic crustal thickness from seismic measurements and rare earth element inversions: Journal of Geophysical Research, v. 97, p. 19683-19715.
